# Metabolite patterns predicting sex and age in participants of the Karlsruhe Metabolomics and Nutrition (KarMeN) study

**DOI:** 10.1371/journal.pone.0183228

**Published:** 2017-08-16

**Authors:** Manuela J. Rist, Alexander Roth, Lara Frommherz, Christoph H. Weinert, Ralf Krüger, Benedikt Merz, Diana Bunzel, Carina Mack, Björn Egert, Achim Bub, Benjamin Görling, Pavleta Tzvetkova, Burkhard Luy, Ingrid Hoffmann, Sabine E. Kulling, Bernhard Watzl

**Affiliations:** 1 Department of Physiology and Biochemistry of Nutrition, Max Rubner-Institut, Karlsruhe, Germany; 2 Department of Quality and Safety of Fruit and Vegetables, Max Rubner-Institut, Karlsruhe, Germany; 3 Institute of Organic Chemistry and Institute for Biological Interfaces 4, Karlsruhe Institute of Technology, Karlsruhe, Germany; 4 Department of Nutrition Behaviour, Max Rubner-Institut, Max Rubner-Institut, Karlsruhe, Germany; Instituto de Investigacion Sanitaria INCLIVA, SPAIN

## Abstract

Physiological and functional parameters, such as body composition, or physical fitness are known to differ between men and women and to change with age. The goal of this study was to investigate how sex and age-related physiological conditions are reflected in the metabolome of healthy humans and whether sex and age can be predicted based on the plasma and urine metabolite profiles.

In the cross-sectional KarMeN (Karlsruhe Metabolomics and Nutrition) study 301 healthy men and women aged 18–80 years were recruited. Participants were characterized in detail applying standard operating procedures for all measurements including anthropometric, clinical, and functional parameters. Fasting blood and 24 h urine samples were analyzed by targeted and untargeted metabolomics approaches, namely by mass spectrometry coupled to one- or comprehensive two-dimensional gas chromatography or liquid chromatography, and by nuclear magnetic resonance spectroscopy. This yielded in total more than 400 analytes in plasma and over 500 analytes in urine. Predictive modelling was applied on the metabolomics data set using different machine learning algorithms.

Based on metabolite profiles from urine and plasma, it was possible to identify metabolite patterns which classify participants according to sex with > 90% accuracy. Plasma metabolites important for the correct classification included creatinine, branched-chain amino acids, and sarcosine. Prediction of age was also possible based on metabolite profiles for men and women, separately. Several metabolites important for this prediction could be identified including choline in plasma and sedoheptulose in urine. For women, classification according to their menopausal status was possible from metabolome data with > 80% accuracy.

The metabolite profile of human urine and plasma allows the prediction of sex and age with high accuracy, which means that sex and age are associated with a discriminatory metabolite signature in healthy humans and therefore should always be considered in metabolomics studies.

## Introduction

The human metabolome is influenced by a number of endogenous factors, such as age, sex, and body mass index (BMI) [1–7], as well as exogenous factors including diet, drugs, physical activity, psychological stress and further environmental factors [8–12]. Among the endogenous factors, age and sex have been addressed most often in literature and seem to have the strongest influence, at least in healthy subjects [13, 14]. A number of metabolites have been reported to differ between older and younger individuals or between men and women. For example, creatinine, tryptophan, histidine or serine have repeatedly been shown to be higher in concentration in urine and/or blood in younger persons, whereas citrate, creatine, glycine, glutamate were described to be higher in older persons [2, 5, 7, 13, 15–18]. In women, menopausal status causes a clear shift in plasma metabolite concentrations [1, 19] and thus may be the underlying cause for apparent age differences. Differences between men and women were suggested to be mainly based on higher plasma and/or urine concentrations of branched-chain amino acids (BCAA) and creatinine in men, whereas several phospholipids, citrate, glycine, hippurate and others were higher in women [2–5, 13, 16, 18, 20–23]. Up to now mechanistic explanations for these observed differences have been scarce.

Most of the early studies were conducted with a rather small number of participants (n = 60–150) that were not very well characterized in terms of diet or health status. Further, biological samples were often analyzed using nuclear magnetic resonance spectroscopy (NMR) as the only analytical method, data analysis was mainly based on principle component analysis (PCA) and partial least squares discriminant analysis (PLS-DA), and thus results have been rather descriptive [2, 5, 16, 18, 22]. More recent studies reporting metabolome variations due to age and/or sex were based on larger cohorts. Metabolite profiles were derived from liquid chromatography (LC)- or gas chromatography-mass spectrometry (GC-MS) analyses, which generally provide a larger coverage of the metabolome than NMR, and more sophisticated univariate and multivariate data analysis methods were applied [3, 6, 7, 13, 15, 23]. Still, these reports are generally restricted to one type of biological sample, i.e. blood or urine, and one type of analytical method, i.e. MS or NMR analysis.

We performed a cross-sectional study [24] to investigate the inherent variation in the human metabolome including healthy male and female participants spanning a wide age range. These participants were thoroughly characterized based on several anthropometric, physiological and functional parameters, including menopausal status in women. Fasting plasma and 24 h urine samples were collected and analyzed using non-targeted comprehensive two-dimensional gas chromatography (GC×GC)-MS, different targeted GC-MS and LC-MS/MS methods as well as ^1^H-NMR. With this multi-platform metabolomics analysis—combining targeted and non-targeted methods—we were able to cover more than 400 analytes in plasma and over 500 analytes in urine from a wide range of chemical classes. The aim of this study was to identify metabolite patterns, i.e. a set of metabolites that in combination and their relation to one another are predictive for sex or age in plasma and 24 h urine of healthy men and women. For this purpose, we performed predictive modelling using three different established machine learning algorithms on the combined data from the different analytical platforms.

## Materials and methods

### Subjects and study design

The Karlsruhe Metabolomics and Nutrition (KarMeN) study is a cross-sectional study that was performed at the Max Rubner-Institut in Karlsruhe, Germany, between 2011 and 2013, and was described in detail in Bub et al. [24].

Briefly, 301 healthy adults, 172 men, 129 women, aged 18–80 y, BMI 17.8–31.4 kg/m^2^, who gave their written informed consent were recruited. Participants were included, if they were free from prevalent diseases, had no history of a chronic disease, were non-smokers, did not take any medication, hormones, or supplements, and were willing and able to perform all the examinations.

Participants were subjected to a standardized examination schedule [24] and visited the study center for a total of three days. Subjects were thoroughly characterized and a number of anthropometric (including height, weight, waist circumference, and body composition), functional (including blood pressure, arterial stiffness, and pulmonary function), and clinical parameters (including blood and urine clinical biochemistry) were determined. Also, resting energy expenditure and cardio-respiratory fitness were assessed. Samples for metabolomics analyses were collected on study day 2.

In addition, the menopausal status in women was determined by anamnestic interview and follicle stimulating hormone (FSH) measurements. For premenopausal women the study days were scheduled such that sample collection on day 2 fell into the luteal phase of their menstrual cycle, since this is the phase of the cycle with the least hormonal variation.

The study was approved by the ethics committee of the State Medical Chamber of Baden-Württemberg, Stuttgart, Germany (F-2011-051) and was in accordance with the 1964 Helsinki declaration and its later amendments. The study was registered at the German Clinical Study Register (DRKS00004890). WHO universal trial number: U1111-1141-7051. Written informed consent was obtained from all individual participants included in the study.

### Samples

On the morning of study day 2, fasting plasma samples were collected by drawing blood into 9 mL ethylenediaminetetraacetic acid (EDTA) plasma tubes (S-Monovette, Sarstedt, Nümbrecht, Germany) from an antecubital vein. Blood was centrifuged at 1850 x g at 4°C and aliquoted into small portions. In addition, serum samples (S-Monovette Z-gel, Sarstedt, Nümbrecht, Germany) were collected for standard clinical biochemistry analyses.

Subjects collected 24 h urine, starting the morning prior to study day 2 up to the morning of study day 2. Collection bottles were kept in cool bags with cooling units throughout. Upon delivery of the 24 h urine samples to the study center, the volume was recorded, 2 x 14 mL were centrifuged at 1850 x g at 20°C and aliquoted into small portions.

All samples were initially frozen at -20°C for one day and then cryopreserved at -196°C until analysis as previously found to be an acceptable procedure [25].

Quality control (QC) samples were prepared by pooling fasting plasma samples and 24 h urine samples, respectively, from KarMeN participants. These QC samples were used for all analytical methods applied.

### Metabolomics analyses

In order to obtain a preferably broad coverage of the metabolome of human biofluids, a number of different targeted and non-targeted analytical methods were applied. This section provides a short overview on the different analytical methods used. A detailed description of the analytical procedures can be found in the S1 File.

#### Untargeted GC×GC-MS analysis of plasma and urine samples

All 24 h urine and fasting plasma samples were analyzed by untargeted GC×GC-MS using a Shimadzu GCMS QP2010 Ultra instrument equipped with a ZOEX ZX2 modulator. The method was originally developed and described for the analysis of urine samples [26], but here was also used for plasma. Urine samples were diluted according to osmolality before analysis in order to reduce matrix effects [27]. Plasma samples were prepared by deproteinization with methanol as well as defatting with tert-butyl methyl ether [28] in order to reduce instrument contamination during the prolonged measurements. With this method a wide range of metabolites can be detected, such as amines, amino acids, organic acids, sugars, sugar alcohols, other polyols etc. For details see section A and B of the supplemental S1 File.

#### Semi-targeted GC-MS analysis of sugar species in urine samples

As some isomeric sugar species cannot be sufficiently resolved with the untargeted GC×GC-MS approach [26] but may play an important role in human metabolism, a complementary targeted GC-MS sugar profiling method was developed for urine samples using a Shimadzu GCMS QP2010 Ultra instrument. Using a Scan-/ selected ion monitoring (SIM)-approach, a higher selectivity and a sufficient sensitivity were achieved. Furthermore, by monitoring common and well-known sugar fragments, e.g., m/z 217, 307, and 361 as well as fragments specific for sugar-related compounds like m/z 292, 333, or 318, known as well as unknown sugar species could be detected. Additionally, some abundant non-sugar compounds always present in urine, such as creatinine, were also captured. This enabled verification of the results of the GC×GC-MS approach. Overall, 66 metabolites, consisting of 40 known sugar species, 15 unknown sugar species, and 11 non-sugar-compounds, were detected with this method. For analytical details see section A and C of the S1 File.

#### Targeted GC-MS analysis of fatty acids in plasma

The chromatographic separation of plasma fatty acids, especially the cis/trans isomers usually requires the application of specialized polar columns and can thus not be done adequately using a standard apolar × medium-polar GC×GC column setup. For this reason, we used the method described by Ecker et al. [29] to determine plasma fatty acids as methyl esters (FAMEs), with minor modifications. Using a GC single quadrupole instrument (Shimadzu GCMS QP2010 Ultra) and a BPX90 column (Trajan Scientific), 48 fatty acids could be determined in plasma. For details see section A and D of the S1 File.

#### LC-MS metabolite profiling using the Absolute IDQ™ p180 kit

Acylcarnitines, amino acids, biogenic amines, phosphatidylcholines and sphingomyelins were determined by LC-MS in fasting plasma samples using the Absolute IDQ™ p180 kit developed by Biocrates AG (Innsbruck, Austria). The 96 well plate kit includes a detailed description for the extraction procedure, instrument settings and quantification software (MetIDQ version 4.5.2). A description of the preparation and quantification process can be found in Romisch-Margl et al. [30]. For chromatographic separation of amino acids and biogenic amines a Zorbax Eclipse XDB-C18 column (3 x 100 mm, 3.5 μm; Agilent, Waldbronn, Germany) equipped with a SecurityGuard™ column (C18, 4.0 x 3.0 mm; Phenomenex, Aschaffenburg, Germany) was used. Phosphatidylcholines and sphingomyelins were analyzed by flow injection analysis (FIA) into the analytical system which was comprised of a Nexera UHPLC system (Shimadzu) coupled to an API QTRAP® 5500 mass spectrometer (AB Sciex, Darmstadt, Germany).

For details regarding reliability of results refer to section E of the S1 File.

#### Targeted LC-MS analysis of methylated amino compounds

A targeted quantification UPLC-MS/MS method for seven amino compounds in plasma, including l-carnitine, choline, and trimethylamine-N-oxide (TMAO), was established [31] using an Acquity UPLC H-Class system coupled to a Xevo TQD triple quadrupole MS (both from Waters, Eschborn, Germany). After protein precipitation and dilution with acetonitrile, plasma samples were separated by Hydrophilic Interaction Liquid Chromatography (HILIC) mode on a BEH Amide column (Waters) using a reversed acetonitrile gradient. Up to two specific fragments of target analytes and deuterated internal standards were monitored using positive electron spray ionization (ESI) in multiple reaction monitoring (MRM) mode. Calibrators and controls were made by spiking plasma samples. Method details can be found in section F of the S1 File.

#### Targeted LC-MS analysis of bile acids

Analyses of 14 bile acids were done from fasting plasma using a 1200 series HPLC system (Agilent, Waldbronn, Germany) coupled with a Q-Trap 3200 mass spectrometer (AB Sciex, Darmstadt, Germany). Samples were prepared by SPE procedure prior to LC-MS/MS analyses using negative ESI and MRM mode as described in detail in Frommherz et al. [32]. Additional information on reliability of results can be found in section G of the S1 File.

#### Untargeted NMR-analysis of plasma and urine samples

All plasma and urine samples were analyzed by 1D-^1^H-NMR spectroscopy. Plasma samples were measured at 310 K on an AVANCE II 600 MHz NMR spectrometer equipped with a ^1^H-BBI probehead and a BACS sample changer (Bruker BioSpin GmbH, Rheinstetten, Germany). Urine samples were analyzed at 300 K on a Bruker 600 MHz spectrometer (either AVANCE III equipped with a ^1^H,^13^C,^15^N-TCI inversely detected cryoprobe or AVANCE II with ^1^H-BBI room temperature probe (Bruker BioSpin GmbH, Rheinstetten, Germany)) equipped with either SampleXpress or BACS sample changer, respectively, as described in Rist et al. [25]. Typically, metabolites that can be detected include organic acids, amino acids, amines, sugars, sugar alcohols, and others. Method details can be found in section H of the S1 File.

#### Standard clinical biochemistry

Calcium, chloride, potassium, sodium, and phosphate concentrations were determined in a 24 h urine specimen. Calcium, chloride, potassium, sodium, phosphate, and also iron concentrations, as well as creatinine, bilirubin, LDL-, HDL-, and total cholesterol, triglycerides, glucose, uric acid, urea, free T3 and free T4 thyroid hormone concentrations were determined in blood serum. Analyses were carried out by the medical laboratory MVZ Labor PD Dr. Volkmann und Kollegen GbR (Karlsruhe, Germany), which is an accredited lab according to DIN EN ISO 15189:2001, using standard analytical procedures. Creatinine was quantified in-house in 24 h urine specimens using a photometric assay based on the Jaffé reaction (DetectX® Urinary Creatinine Detection Kit; Arbor Assays, Ann Arbor, Michigan, USA). Total urinary nitrogen was quantified by the Kjeldahl method. FSH was quantified in blood serum by an enzyme-linked immunosorbent assay (ELISA) (Human FSH ELISA, BioVendor, Brno, Czech Republic). Urine osmolality was directly determined by freezing-point depression, using a micro-osmometer (Advanced Micro-Osmometer model 3MO, Advanced Instruments, Norwood, MA, USA).

### Data processing

#### (GC×)GC-MS

GC×GC-MS raw data files were processed by untargeted alignment by in-house developed R-modules, as described recently [33]. Signal intensity drift, i.e. intra- and inter-batch effects occurring during the 4–5 week measurement period were corrected by means of regularly injected QC samples [34–36]. For the data of the semi-targeted GC-MS analysis of sugar species in urine, an automatic method for integration was prepared using the Postrun Analysis feature of GCMSsolution (v 4.1.1.). The parameters used to determine area and height of peaks and for identification are given in section C of the S1 File. Peaks were integrated according to target and reference ions, their ratios as well as deviation allowance for these ratios. Results of automatic integration were checked manually (see section C of the S1 File). An excel table with integrated peak areas of the chosen substances was created for further data processing.

#### LC-MS metabolite profiling (Absolute IDQ™ p180 kit)

To analyze the samples of the entire study, five Absolute IDQ™ well plates were used. To account for possible batch effects between the plates, data normalization as described by the manufacturer’s user manual was applied based on the pooled QC samples which were extracted and measured ten times on each well plate in between the study samples. The efficacy of the procedure was verified by comparing PCA plots and coefficients of variation before and after plate correction.

#### NMR

All spectra were automatically phased with the Bruker AU program apk0.noe. Using the program AMIX 3.9.14 (Bruker BioSpin GmbH, Rheinstetten, Germany) plasma spectra were then referenced to the ethylenediaminetetraacetic acid (EDTA) signal at 2.5809 ppm and bucketed graphically, such that buckets wherever possible contained only one signal or group of signals and no peaks were split between buckets. Urine spectra were resampled to bring them to a uniform frequency axis. Then, spectra were aligned by “correlation optimized warping” [37] and bucketed using an in-house developed software based on Python, again, intended to define buckets that contain only one signal or group of signals and not splitting peaks between buckets whenever possible. Resulting bucket tables were used for statistical analyses and machine learning algorithms after normalizing to osmolality in case of urine data.

### Data analysis

Data of the different analytical platforms were integrated into a combined data matrix, consisting of 301 samples and > 1000 analytes (including knowns and unknowns). Analytes with a detected frequency lower than 75% in the study samples were eliminated from the data matrix prior to statistical analysis. Non-detected values were replaced by values corresponding to 1/10 x limit of quantitation (LOQ) in targeted methods, where no limit of detection (LOD) was determined; 1/2 x LOD in methods, where LOD was determined/available; or 1/2 x minimal intensity for non-targeted MS-based methods.

The analytes were arranged in columns of this common data matrix, which were mean centered and scaled by standard deviation prior to analysis. This resulting matrix was used as input for three different prediction models: support vector machine (SVM) with linear kernel, generalized linear model net (glmnet), and PLS. Three algorithms were used in order to support the interpretation of the results, assuming that metabolites appearing as important in different algorithms are biologically relevant. The prediction performance of these models is dependent of model specific hyperparameters which have to be optimized. For example, SVM uses a cost parameter C that controls the trade-off between complexity of the decision function and training error. The parameters α and λ are tuned in glmnet, and the number of components (ncomp) in PLS. In order to find the optimal value for the hyperparameter, a grid search in conjunction with a nested 5x10-fold cross validation scheme [38] was applied, and the average of the resulting 50 values was used in the final model. This way, in PLS the number of components included in the model ranged between 2 and 7, depending on the question (sex, age, menopause) and matrix (plasma, urine). When the model was used to predict sex or menopausal status, the classification accuracy was assessed. For the continuous model of age the root mean squared error (RMSE) and R^2^ were calculated to estimate performance of the predictions. Details on nested cross-validation can be found in section I of the S1 File.

For the prediction of sex, due to the relatively low number of young females compared to young men, only participants in the age range of 36–80 were included. In this age range the number of males and females is comparable (n = 99 and n = 101, respectively).

Each prediction model yields a metabolite pattern, i.e. a combination of analytes that is important for the correct prediction. In case of linear SVM and glmnet negative and positive weights occur, e.g. favoring the male or female class, respectively. In case of PLS positive and negative weight are distributed over multiple components and can only be summarized into a single value by summing up the squared weights from the different components. For the presentation of results, analytes were assigned a rank for each algorithm according to their weight, the ranks of the three algorithms were averaged, and analytes sorted according to mean rank.

Identification of unknown substances from non-targeted analyses that are important for the prediction of sex or age was performed by comparison with databases, as described in Weinert et al. and Egert et al. [26, 33] for GC×GC-MS or with the Chenomx NMR Suite 8.1 (Chenomx, Edmonton, Canada) for NMR.

## Results

### Basic characteristics of KarMeN study participants

Basic characteristics of the KarMeN study participants as well as selected anthropometric, physiological and functional parameters assessed are listed in **Table 1**.

**Table 1:** Basic characteristics of KarMeN study participants.

	Female	Male	Total
	n = 129^a^	n = 172	n = 301
**Age (y)**	51.7 ± 15.0^b^	44.4 ± 17.9	47.5 ± 17.1
**BMI (kg/m^2^)**	23.2 ± 2.9	24.4 ± 2.7	23.9 ± 2.9
**Body fat (%)**	34.8 ± 6.8	23.9 ± 6.6	28.5 ± 8.6
**BP**^**c**^ **sys (mmHg)**	121 ± 18	128 ± 14	125 ± 16
**BP dias (mmHg)**	83.8 ± 12.4	84.8 ± 10.6	84.4 ± 11.4
**Basal metabolic rate (kcal/d)**	1194 ± 127	1574 ± 191	1411 ± 251
**Total serum cholesterol (mg/dL)**	209 ± 39.5 (n = 128)	191 ± 45.1	199 ± 43.6 (n = 300)
**Serum glucose (mg/dL)**	84.9 ± 7.5 (n = 128)	86.6 ± 8.2	85.9 ± 8.0 (n = 300)
**Serum insulin (μIU/mL)**	9.64 ± 6.94 (n = 127)	10.25 ± 4.50	9.99 ± 4.27 (n = 299)

^a^ with n = 56 in pre- and n = 73 in post-menopausal state at time of sampling and examination

^b^ Data are given in mean ± SD.

^c^ Abbreviations: BP, blood pressure; sys, systolic; dias, diastolic

### Metabolomics data from the multi-platform approach

All plasma samples were analyzed by GC×GC-MS, GC-MS, LC-MS/MS, NMR, and classical clinical biochemistry. After quality checks and data filtering, 442 analytes were included in the metabolomics data analyses. Of these, 174 were derived from targeted analyses and thus known a priori. Of the detected analytes from non-targeted analyses, approximately 40% could be identified or putatively annotated by comparison with databases. For 24 h urine samples, in addition to classical clinical biochemistry analyses, metabolomics analyses were performed using GC×GC-MS, GC-MS, and NMR, yielding 531 analytes after data filtering. Targeted analyses contribute 57 a priori known metabolites, whereas from the non-targeted analyses approximately 28% of analytes could be identified or putatively annotated. The complete dataset is shown in S1 Table.

### Prediction of sex

In order to determine if the metabolite profiles of the KarMeN study participants are associated with sex, predictive modelling was performed on the combined data matrix including analytes form all analytical methods used. Prediction of sex was possible from plasma metabolite profiles with generally more than 95% accuracy, whereas accuracy of prediction of sex from urine metabolite profiles was possible with about 90%, with PLS yielding the highest accuracy for both sample matrices (**Table 2**). The combination of plasma and urine profiles did not improve the accuracy of prediction.

**Table 2:** Prediction accuracy of sex of the KarMeN study participants based on metabolite profiles in plasma and urine using different algorithms.

Matrix	Algorithm	Accuracy % (total) n = 200	Accuracy % (men) n = 99	Accuracy % (women) n = 101
**Plasma**	SVMlinear	96.7	95.9	97.6
	glmnet	95.9	95.9	96.0
	PLS	97.3	96.1	98.5
**Urine**	SVMlinear	90.3	92.0	88.5
	glmnet	90.5	89.4	91.5
	PLS	90.5	93.5	87.4
**Plasma + Urine**	SVMlinear	95.8	95.0	96.6
	glmnet	95.8	95.3	96.3
	PLS	97.2	97.9	96.4

**Fig 1:**
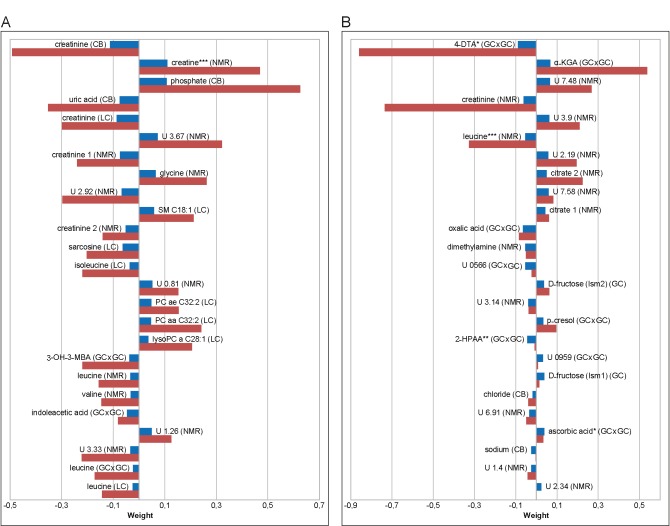
Metabolite patterns for the prediction of sex. Top 25 metabolites important for the correct prediction of sex of the KarMeN study participants in all algorithms applied on plasma (A) and 24 h urine (B) metabolite profiles. Positive and negative weights favor female and male class, respectively. Patterns are shown for linear SVM (blue bars) and glmnet (red bars) only, since PLS only yields positive values. Metabolites are sorted according to “mean rank” of all three algorithms. Analytical methods from which metabolites stem are denoted in parentheses, with CB, clinical biochemistry; GC, GC-MS; GC×GC, GC×GC-MS; LC, LC-MS; NMR, nuclear magnetic resonance. * Tentatively identified using the NIST2011 library solely based on mass spectral similarity. ** Identified using the FIEHN library based on mass spectral similarity and retention index. *** Signal possibly includes other metabolites. Abbreviations: U, unknown; 3-OH-3-MBA, 3-hydroxy-3-methylbutyric acid; 4-DTA, 4-deoxythreonic acid; α-KGA, α-ketoglutaric acid; 2-HPAA, 2-hydroxyphenylacetic acid.

Metabolites most important for the correct prediction of sex from plasma or 24 h urine that occurred in the patterns derived from all three algorithms are shown in Fig 1 and include metabolites from all analytical platforms used. Metabolites with positive or negative weights in the metabolite pattern tend to show higher concentrations in women or men, respectively (S1 Fig), although this does not necessarily have to be correct for all metabolites included in the patterns. Most of these metabolites detected in plasma could be identified and include creatinine, uric acid, sarcosine and the BCAA leucine, isoleucine, or indoleacetic acid, which all have negative weights, i.e. generally show higher concentrations in men. Creatine, phosphate, glycine, sphingomyelin (SM) C18:1, and several phosphatidylcholines, on the other hand, have positive weights, i.e. tend to show higher concentrations in women. The two urine metabolites with the highest mean ranks could be identified as 4-deoxythreonic acid (higher in men) and α-ketoglutaric acid (higher in women). Other urinary metabolites contributing to the correct prediction of sex included creatinine, leucine, dimethylamine, 2-hydroxyphenylacetic acid, chloride, and sodium with higher concentrations in men; and citrate, fructose, and *p*-cresol with higher concentrations in women (Fig 1). Unfortunately, several of the urinary analytes important for the prediction of sex could not be identified yet.

### Prediction of age

In order to determine whether the metabolite profiles of the KarMeN study participants are associated with age, predictive modelling was performed on plasma and urine metabolite profiles using three different algorithms. Since metabolite profiles of humans are different between sexes, these predictions for age were performed separately for men and women.

#### Prediction of age in men

For men, all algorithms used showed clear associations of metabolite profiles with age (Table 3), with PLS generally showing the best prediction (R^2^ approx. 0.7).

**Table 3:** Prediction of chronological age of the KarMeN study participants based on metabolite profiles in plasma and urine using different algorithms.

		Men (n = 172)		Women (n = 129)	
**Plasma**	**Algorithm**	**RMSE**	**R**^**2**^	**RMSE**	**R**^**2**^
	SVMlinear	9.09	0.729	9.44	0.58
	glmnet	9.33	0.713	9.60	0.559
	PLS	8.39	0.773	9.19	0.603
**Urine**	**Algorithm**	**RMSE**	**R**^**2**^	**RMSE**	**R**^**2**^
	SVMlinear	10.98	0.607	10.25	0.482
	glmnet	10.79	0.619	10.88	0.418
	PLS	9.79	0.687	9.37	0.575
**Plasma and Urine**	**Algorithm**	**RMSE**	**R**^**2**^	**RMSE**	**R**^**2**^
	SVMlinear	8.73	0.75	9.11	0.608
	glmnet	9.06	0.732	9.72	0.553
	PLS	8.31	0.776	9.02	0.611

**Fig 2:**
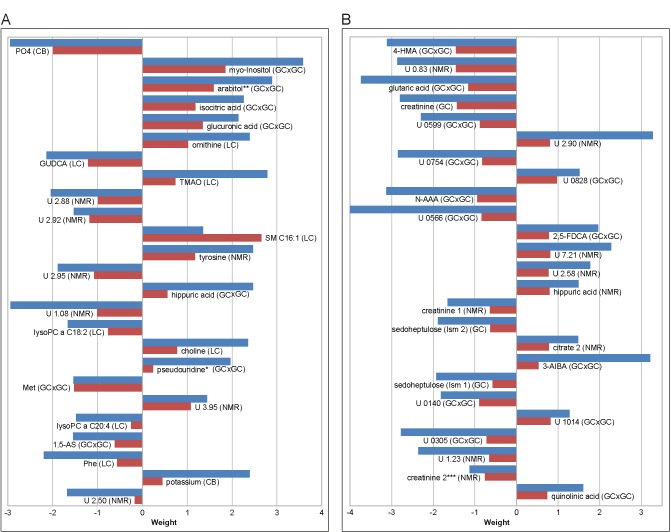
Metabolite patterns for the prediction of age in men. Top 25 metabolites important for the correct prediction of age of the male KarMeN study participants in all algorithms applied on plasma (A) and 24 h urine (B) metabolite profiles. Positive and negative weights favor older and younger age, respectively. Patterns are shown for linear SVM (blue bars) and glmnet (red bars) only, since PLS only yields positive values. Metabolites are sorted according to “mean rank” of all three algorithms. Analytical methods from which metabolites stem are denoted in parentheses, with CB, clinical biochemistry; GC, GC-MS; GC×GC, GC×GC-MS; LC, LC-MS; NMR, nuclear magnetic resonance. * Tentatively identified using the NIST2011 library solely based on mass spectral similarity. ** Identified using the FIEHN library based on mass spectral similarity and retention index. *** Signal possibly includes other metabolites. Abbreviations: U, unknown; PO4, phosphate; Met, L-methionine; 1,5-AS, 1,5-anhydro-D-sorbitol; Phe, phenylalanine; 4-HMA, 4-hydroxymandelic acid; N-AAA, N-acetylaspartic acid; 2,5-FDCA, 2,5-furandicarboxylic acid; ism, isomer; 3-AIBA, 3-aminoisobutyric acid.

The most important metabolites for the correct prediction of age that occur in the metabolite patterns of all three algorithms used are shown in Fig 2. Again, analytes from all applied analytical platforms are contained in this list. Metabolites with positive or negative weights in the metabolite pattern tend to show higher concentrations in older or younger men, respectively (S2 Fig). Most of the metabolites detected in male plasma could be identified and include phosphate, glycoursodeoxycholic acid (GUDCA), lysophosphatidylcholine (lysoPC) a C18:2, and l-methionine, which show negative weights, i.e. generally higher concentrations in younger men. In contrast, *myo*-inositol, arabitol, isocitric acid, glucuronic acid, l-ornithine, TMAO, SM C16:1, l-tyrosine, hippuric acid, choline, pseudouridine, and potassium show positive weights and therefore tend to have a higher concentration in older men (Fig 2). In urine samples of male participants, many of the analytes important for correct prediction of age could not be identified. Of the known metabolites, 4-hydroxymandelic acid, glutaric acid, creatinine, N-acetylaspartic acid, and sedoheptulose show higher concentrations in younger men, whereas 2,5-furandicarboxylic acid, hippuric acid, citric acid, 3-aminoisobutyric acid, and quinolinic acid show a higher concentration in older men (Fig 2).

#### Prediction of age in women

For women, the associations of metabolite profiles with age were less strong than for men (Table 3) with PLS again yielding the best prediction (R^2^ approx. 0.6). The most important mean rank metabolites for the correct prediction of age are shown in S3 Fig. Again, analytes from all applied analytical platforms are contained in the ranking, and the positive or negative weights tentatively indicate higher concentrations of metabolites in older or younger women, respectively.

In female plasma samples many metabolites important for the correct prediction of age could be identified and include ornithine, choline, pseudouridine, hippuric acid, meso-erythritol, potassium, phenylalanine, SM(OH)C14:1, SM(OH)C16:1, cholesterol, d-glucuronic acid, glucose, and phosphatidylcholine (PC).aa.C32:2, which tend to be higher in older women, whereas PC.ae.C44:5, isoleucine, aspartic acid, malic acid, and tryptophan tend to be higher in younger women (S3 Fig). Also, many of the analytes important for correct prediction of age from female urine samples could be identified. The known metabolites that tend to be higher in younger women include sedoheptulose, N-acetyl-l-aspartic acid, glutaric acid, uracil, succinic acid, 1,5-anhydro-d-sorbitol, creatinine, erythronolactone, and tiglylglycine. On the other hand, 2-*O*-methylascorbic acid, formic acid, 2,5-furandicarboxylic acid, 4-hydroxyphenyllactic acid, and tartaric acid tend to be higher in older women (S3 Fig).

#### Metabolites important for prediction of age in men and women

Several metabolites were found to be associated with age in both sexes and could therefore be described as sex-independent markers of age. They include ornithine, hippuric acid, choline, pseudouridine, glucuronic acid, phenylalanine, potassium in plasma and glutaric acid, creatinine, N-acetylaspartic acid, 2,5-furandicarboxylic acid, sedoheptulose, and several unknown metabolites in urine (Fig 2 and S3 Fig).

#### Classification of menopausal status in women

Prediction of age in women was less accurate than prediction of age in men, and concentrations of many metabolites showed a discontinuous trajectory with age, with a sudden increase around the age of 50 (S4 Fig). Thus, the prediction of age in women was also performed in a categorical manner for pre- and post-menopausal women using the same classification analyses as for the prediction of sex.

Based on plasma metabolome data, prediction of menopausal status was possible with a high accuracy of about 88%, whereas prediction based on urine metabolome data was possible with up to 85% accuracy, with PLS being the algorithm producing the best results in both cases (S2 Table). Combining the data from plasma and urine samples increased the accuracy of prediction to about 90%, where SVM showed the best performance (S2 Table).

Of the plasma metabolites most important for the correct prediction of menopausal status only few were identical to the ones important for the prediction of continuous age in women, and include ornithine, choline, glucuronic acid, and cholesterol. Other plasma metabolites important for the correct classification of women according to their menopausal status include creatinine, serine, *myo*-inositol, carnitine, and others (S5 Fig). Important metabolites in urine were largely identical to the ones important for prediction of continuous age in women, such as sedoheptulose, 1,5-anhydro-d-sorbitol, and uracil. Other urinary metabolites include 4-pyridoxic acid, glycine, ribose, and aminomalonic acid (S5 Fig).

## Discussion

### KarMeN study and multi-platform approach

The participants of the KarMeN study were very well characterized, including anthropometric, physiological and functional measures. Samples were generated according to established SOPs and under very controlled conditions, with plasma samples collected in the fasting state and a 24 h urine sample collected on the day prior to and up to blood sampling. We focused on healthy participants in a normal to moderately overweight weight range, in order to minimize any interference with diseases, medication or metabolic anomalies. Further, menopausal status in women was assessed. Since the use of oral contraceptives has been described to alter the plasma metabolome [4], hormonal treatment or supplement use was not allowed for participants in order to reduce metabolic variation due to hormones or supplements.

Care was also taken to control every step along the metabolomics pipeline in the best possible way from study design to sample collection, sample storage, sample analysis, and data analysis. Therefore, although the study included only 301 participants and the study design was cross-sectional, the data constitutes valuable information for validation investigations.

Previous studies investigating the impact of age and sex on the human metabolome have based their findings mostly on only one body fluid, and applied mainly one analytical method [2, 5, 16, 18, 22]. We investigated both plasma and urine and combined several different analytical methods with targeted and non-targeted approaches in order to assure a broad coverage of the metabolome. In this way, after quality control and filtering of the data, we detected more than 400 analytes in plasma and more than 500 analytes in urine from many different chemical classes. Owing to the fact that some metabolites are detected with more than one method and that some metabolites may yield more than one signal in non-targeted methods, the number of unique metabolites will be considerably less. Especially in NMR spectroscopy one compound can produce many signals in various regions of the spectra, resulting in several variables in non-targeted analysis. In addition, automatic alignment of peaks in NMR spectra of urine is difficult and can yield different results, depending on the alignment method applied as reviewed by Vu and Laukens [39]. The fact that some metabolites are detected by several methods, however, could be used as a quality measure of our analyses since correlations of these metabolites between methods was very good in most cases. Therefore, this multi-platform approach is clearly a strength of our study allowing to correlate metabolite concentrations or intensities between different analytical techniques, to judge the quality of the data, and to identify unknowns [40].

Data analysis was done by multivariate methods: three different machine learning algorithms that are suited for analysis of large-scale metabolomic datasets were used to perform cross-validated predictive modelling. This enabled prediction of sex or age of “unknown” samples, and yielded metabolite patterns, i.e. combinations of metabolites that are important for the correct prediction. Since with these methods the coefficient (weight or “importance value”) of one metabolite depends on all others, a single importance value cannot be interpreted outside this context, and even the signs of the importance values do not necessarily show the correct concentration trend for all metabolites. For the discussion, however, metabolites that were important in all three algorithms were considered biologically relevant for the respective questions and are discussed here further (see also S3 Table).

### Association of metabolite profiles with sex

Using different machine learning algorithms, it was possible to correctly predict the sex of the study participants with > 95% accuracy from plasma and > 90% accuracy from urine metabolite profiles. Metabolites that are important for correct prediction from plasma include creatinine detected by different analytical methods, lipids (mainly phospholipids), and amino acids (S3 Table). Especially BCAA and their metabolites seem to play a role in the difference between men and women, showing higher concentrations in male plasma. This has been shown before in adolescents [22, 41] and adults [3, 4, 23, 42–45] and is assumed to be caused by the larger muscle mass in men [46] or a higher protein intake of men compared to women, as a direct association between the intake and plasma levels of BCAA has been shown [47–49]. As described previously in the context of sex differences [3, 4, 6, 50], also in our study several lipid metabolites in plasma were important for the prediction of sex, which were all higher in women. Sarcosine has to the best of our knowledge not been described in the context of sex before. Since it is present in food and can also be formed as an intermediate in the metabolism from choline to glycine, the difference in plasma sarcosine concentrations between men and women could indicate different dietary habits [51]. The intermediates in this pathway, choline, betaine, dimethylglycine, and sarcosine all tend to be higher in men. Glycine, however, tends to be higher in women which is in agreement with previous reports [3, 4, 23]. Therefore, the role of plasma sarcosine in the prediction of sex remains speculative.

Unfortunately, many of the metabolites that are important for the prediction of sex from urine metabolite profiles could not yet be identified. Some of the ones that could be annotated or were derived from targeted analyses have been described before to differ between men and women, such as creatinine [2, 5, 16, 20, 52], which is higher in men and known to be determined by muscle mass, or citrate [2, 5, 16, 20, 53, 54], which is higher in women. The reason behind this is largely speculative. Excretion of citrate is determined in part by the rate of intracellular citrate metabolism [55]. Since another intermediate of the citric acid cycle, α-ketoglutaric acid, is also higher in women, this may hint at a general difference in citric acid cycle turnover between men and women. However, at least in skeletal muscle no sex differences in activities of the enzymes of the citric acid cycle have been found, and the capacity for acetyl-CoA flux through the citric acid cycle appears to be similar in men and women [56].

The metabolite with the highest importance value for the prediction of sex from urine metabolite profiles, 4-deoxythreonic acid, has not been mentioned in literature in the context of sex differences before. It is known to be present in urine [57, 58], and has been described as a metabolite of l-Threonine [59]. Its function, however, is yet unknown. In addition, we found several other metabolites that are important for the prediction of sex that have not been described in this context before (S3 Table).

Although we cannot explain the functional role of the described metabolites with this study, it is easy to imagine that the hormonal differences between men and women, leading among other effects to different body composition, and behavioral or life-style differences are the underlying cause for many of the observed metabolite differences.

One limitation of our study is that we were not able to recruit an adequate number of young females due to exclusion of hormonal contraceptive use. Therefore, prediction models for sex are based on participants ranging from 36 to 80 years of age. Also, due to the fact that we did not further explore analytes that occurred in less than 75% of all study samples, we may have missed metabolites that are specific to one sex.

Based on our results and observations in other studies, we argue that sex has to be taken into account as a potentially confounding factor for all metabolomics analyses that are based on study populations including men and women.

### Association of metabolite profiles with age

Since sex has a large influence on the metabolome, associations of metabolite profiles with age were investigated separately for men and women. Employing different machine learning algorithms, it was possible to predict the chronological age of the study participants in a continuous model based on metabolite profiles from plasma or urine for men and women. However, associations with urine were weaker than with plasma, and for women they were weaker than for men. The latter could be caused by the lower number of women than men or due to menopausal effects.

It has been reported before [1, 19] and is in line with our own data that concentrations of some metabolites in women do not change continuously with age, but show a sudden increase or decrease in concentration around the age of 50 that is associated with menopause. For men, metabolite concentration changes with age occur continuously starting at 30 or 40 years of age (S4 Fig). Therefore, we also calculated a categorical model for women, where age groups were formed according to their menopausal status. Prediction of age category as defined by menopausal status was possible with high accuracy based on plasma and urine metabolite profiles. Interestingly, in plasma mainly other metabolites were important for the prediction of age categories, i.e. menopausal status, than for continuous age prediction, whereas in urine important metabolites for age prediction were largely the same in both models (S3 and S5 Figs). This is most likely due to the fact that the models for continuous age prediction selected for metabolites that change linearly with age, whereas metabolites important for prediction of menopause may show non-linear trajectories. However, prediction scores for classification of menopausal status and the continuous age model are highly correlated (Spearman’s ρ = -0.89 for plasma and -0.84 for urine), and therefore likely describe the same association.

Generally, as reported by others [1, 7, 18, 19, 42, 50, 60], we found that several amino acids, lipids, and organic acids in plasma are important for the prediction of age in women, with ornithine, choline, hippuric acid, cholesterol, glucuronic acid, and glucose occurring in metabolite patterns for chronological age and menopause (S3 Table). Interestingly, all these metabolites have higher concentrations in older and post-menopausal women, respectively. Most of them have been described before in the context of age [1, 16, 18, 61], although urinary hippuric acid was found to be elevated in younger persons [16]. However, choline and glucuronic acid have, to our knowledge not been reported before. Since a number of phospholipids show higher concentrations in older women, it is plausible that choline is also increased in this context. It should be mentioned, however, that the concentration is only marginally higher in older women, and some phospholipids show lower concentrations in older women. Therefore this assumption is purely speculative. Further metabolites contained in the pattern for continuous age in women, that have not been reported in this context, include pseudouridine, potassium (higher in older women), and malic acid (higher in younger women).

Most metabolites in urine that were found to be important for the prediction of age in women have not been reported in this context before with a few exceptions. N-acetyl-aspartic acid was found by Thévenot et al. to be associated with age, but also with sex and BMI [6]. The sugar alcohol 1,5-anhydrosorbitol was found to be decreasing with age in plasma [62], which could explain our observation of lower concentrations in urine in older women. Creatinine in urine decreases with age in our study, which is in line with other studies [2, 5, 16] and could possibly be explained by an age-related decline in lean body mass or renal function. Most other known metabolites contained in the pattern have not been reported as being associated with age. Of those, sedoheptulose was the most important metabolite for the prediction of chronological age and menopausal status. It is a sugar with 7 C-atoms that is an intermediate in the pentose phosphate cycle. Although its presence in urine has been described almost 50 years ago [63], so far it has only been discussed in the context of enzyme deficiencies [64, 65], but not in healthy humans.

Sedoheptulose was also important for the correct prediction of age in men, but with a lower rank than in women (S3 Table). Further metabolites were important for the prediction of age in men and women, such as creatinine and N-acetylaspartic acid, which have been discussed above, but also glutaric acid and 2,5-furandicarboxylic acid, which to our knowledge have not been reported to be associated with age. The concentration of glutaric acid is higher in younger men and women. Since it is a metabolite of lysine degradation, this could again hint at a higher protein intake and a higher supply with essential amino acids, respectively, in younger compared to older participants. 2,5-Furandicarboxylic acid has been described as being present in urine in the early 1970ies by Pettersen and Jellum [66]. They also suggested that this metabolite is derived from exogenous origin, such as furan derivatives in food that was prepared by strong heat treatment [66] again pointing at potentially varying dietary intakes in men and women of different ages.

The urinary metabolite with the highest importance value for the prediction of age in men was 4-hydroxymandelic acid with higher concentrations in younger men. Unfortunately, not much has been reported about this molecule. It has been found as a metabolite in human urine after consumption of a polyphenol mix from red wine and red grape juice extracts [67], suggesting differences in nutrition habits between younger and older men.

When looking at plasma metabolites contained in the metabolite pattern for age of men, analogous to the pattern in women, mainly lipids, amino acids, and organic acids are important (S3 Table). Several metabolites are present in the metabolite pattern for age of men and women, including ornithine, hippuric acid, choline, pseudouridine, glucuronic acid, phenylalanine, and potassium, with phenylalanine being the only metabolite that shows opposite trends in men and women. Other metabolites are specific to men, such as phosphate, GUDCA, lysoPC C18:2, methionine, which are all higher in younger men, or *myo*-inositol, arabitol, isocitric acid, TMAO, and others (higher in older men).

One has to keep in mind that our continuous models were based on chronological age but not on biological age as suggested by Hertel et al. [68], whereas menopausal status may represent biological/physiological age to some extent. Also the described results are observations from a cross-sectional study with samples taken in the fasting, i.e. steady state. Therefore, it is not possible to draw any conclusions on the mechanisms behind the observed associations, since they are most likely a combination of endogenous processes related to aging and exogenous life-style differences, such as dietary habits, physical activity etc.

In general, we listed and discussed metabolites only if they had high ranks in all three algorithms. This way we may have missed some metabolites that are important for the prediction of sex or age in only one or two algorithms, but we concluded that metabolites with high ranks in all three algorithms may have a high biological relevance and are not method-specific.

As elaborated above, many of the metabolites found in this study to be important for the prediction of sex or age have been reported multiple times in different studies. Therefore, these metabolites seem to be reliable and robust markers and thus likely also biologically relevant, since study participants, analytical methods, and statistical methods vary considerably between these studies. While most other studies looked at significant differences in metabolite concentrations between sexes or age groups, our predictive modelling approach identified metabolite patterns that are important for the correct prediction of sex or age, i.e. a set of metabolites that in combination and in their relation to one another are associated with sex or age. If metabolites are found to be associated with sex or age in both approaches, this may indicate their fundamental functions in the metabolism with respect to sex and age.

In addition, due to the wide analytical coverage based on different analytical techniques and the predictive modelling approach we found several metabolites that have not been reported before to be associated with sex or age. Nevertheless, these may also be relevant in other study populations.

Based on our results and observations in other studies [5–7], we argue that age has to be considered as a potentially confounding factor for metabolomics analyses. In addition we and others [1, 19] found that age-trajectories of many metabolites are sex-dependent. This means that also age-sex-interactions have to be included in statistical modeling, which can have important implications for the statistical methodology in the field of metabolomics.

## Conclusions

The clear associations of metabolite patterns of the participants of the KarMeN study with sex and age demonstrate that the human urine and plasma metabolome varies with age and differs between sexes. Using different machine learning algorithms, it was possible to predict sex and age from metabolite profiles of plasma and urine. Due to the fact that we employed a wide spectrum of analytical methods, that the participants of this study were very well characterized, and sampling and examinations were performed in very standardized conditions, several metabolites were found to be important for the correct prediction of sex or age that have not been described before. Other metabolites identified as being important for the prediction of sex or age, have been reported previously. This suggests that these are robust markers for sex or age in healthy humans. Several metabolites were found to be associated with age in men and women. Thus, they could potentially be considered general markers of age. Due to the associations of sex and age with the metabolome and the different age-trends in men and women, we recommend to include not only sex and age but also sex-age-interactions in statistical data analyses of metabolomics studies.

## Supporting information

S1 Fig Boxplots of selected metabolites contained in metabolite patterns important for prediction of sex. (PDF)

S2 Fig Concentrations of selected metabolites contained in metabolite patterns important for prediction of age in male study participants versus age. (PDF)

S3 Fig Metabolite patterns for the prediction of age in women. (PDF)

S4 Fig Heatmap of metabolites from Biocrates platform, sorted according to sex and age. (PDF)

S5 Fig Metabolite patterns for the prediction of menopausal status in women. (PDF)

S1 Table Dataset including metabolite data and metadata. (XLSX)

S2 Table Prediction of menopausal status in female study participants. (PDF)

S3 Table Summary of metabolite patterns. (XLSX)

S1 File Additional methods. (DOCX)

## Abbreviations

BMI: body mass index
BCAA: branched-chain amino acids
PLS: partial least squares
LC: liquid chromatography
GC: gas chromatography
GC×GC: comprehensive two-dimensional gas chromatography
lysoPC: lysophophatidylcholine
MS: mass spectrometry
NMR: nuclear magnetic resonance
SVM: support vector machine
glmnet: generalized linear model net
KarMeN: Karlsruhe Metabolomics and Nutrition
FSH: follicle stimulating hormone
QC: quality control
TMAO: trimethylamine-N-oxide
RMSE: root mean squared error
GUDCA: glycoursodeoxycholic acid
SM: sphingomyelin
PC: phosphatidylcholine
