# Supplementary material for: Metabolite patterns predicting sex and age in participants of the Karlsruhe Metabolomics and Nutrition (KarMeN) study
Source: PLoS One. 2017 Aug 16;12(8):e0183228. doi: 10.1371/journal.pone.0183228 (PMC5558977; doi:10.1371/journal.pone.0183228)
Supplement: S1 Fig — (PDF) [file pone.0183228.s001.pdf]

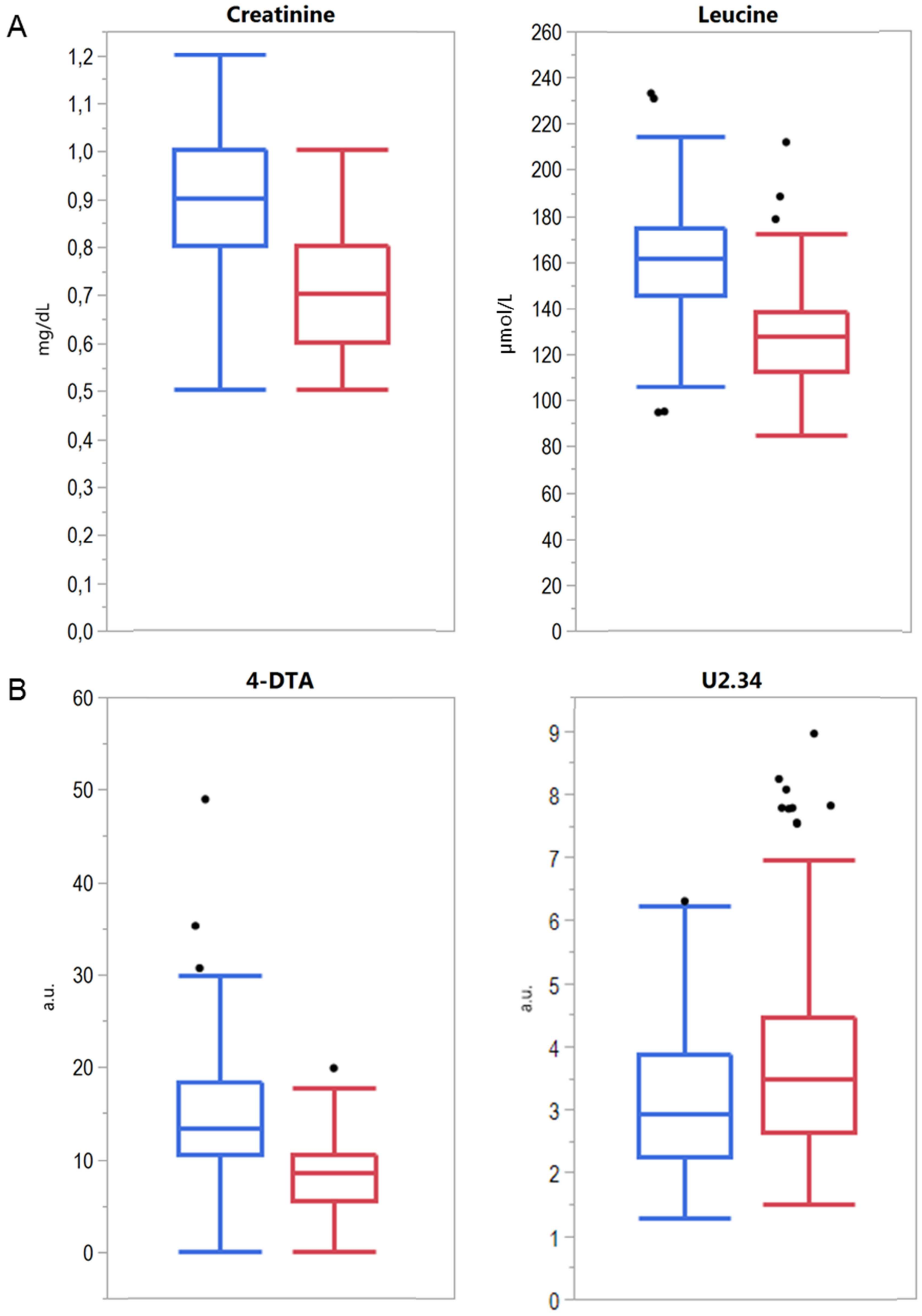

S1 Fig.: Boxplots of selected metabolites contained in metabolite patterns important for prediction of sex. Boxplots are shown for concentrations of (A) plasma and (B) urine metabolites with mean rank 1 (left panel) and 25 (right panel) for men (blue) and women (red). Boxplots are drawn for illustrative purpose only. 4-DTA, 4-deoxythreonic acid; U2.34, unknown NMR analyte around 2.34 ppm; a.u., arbitrary units.
