# Supplementary material for: Metabolite patterns predicting sex and age in participants of the Karlsruhe Metabolomics and Nutrition (KarMeN) study
Source: PLoS One. 2017 Aug 16;12(8):e0183228. doi: 10.1371/journal.pone.0183228 (PMC5558977; doi:10.1371/journal.pone.0183228)
Supplement: S2 Fig — (PDF) [file pone.0183228.s002.pdf]

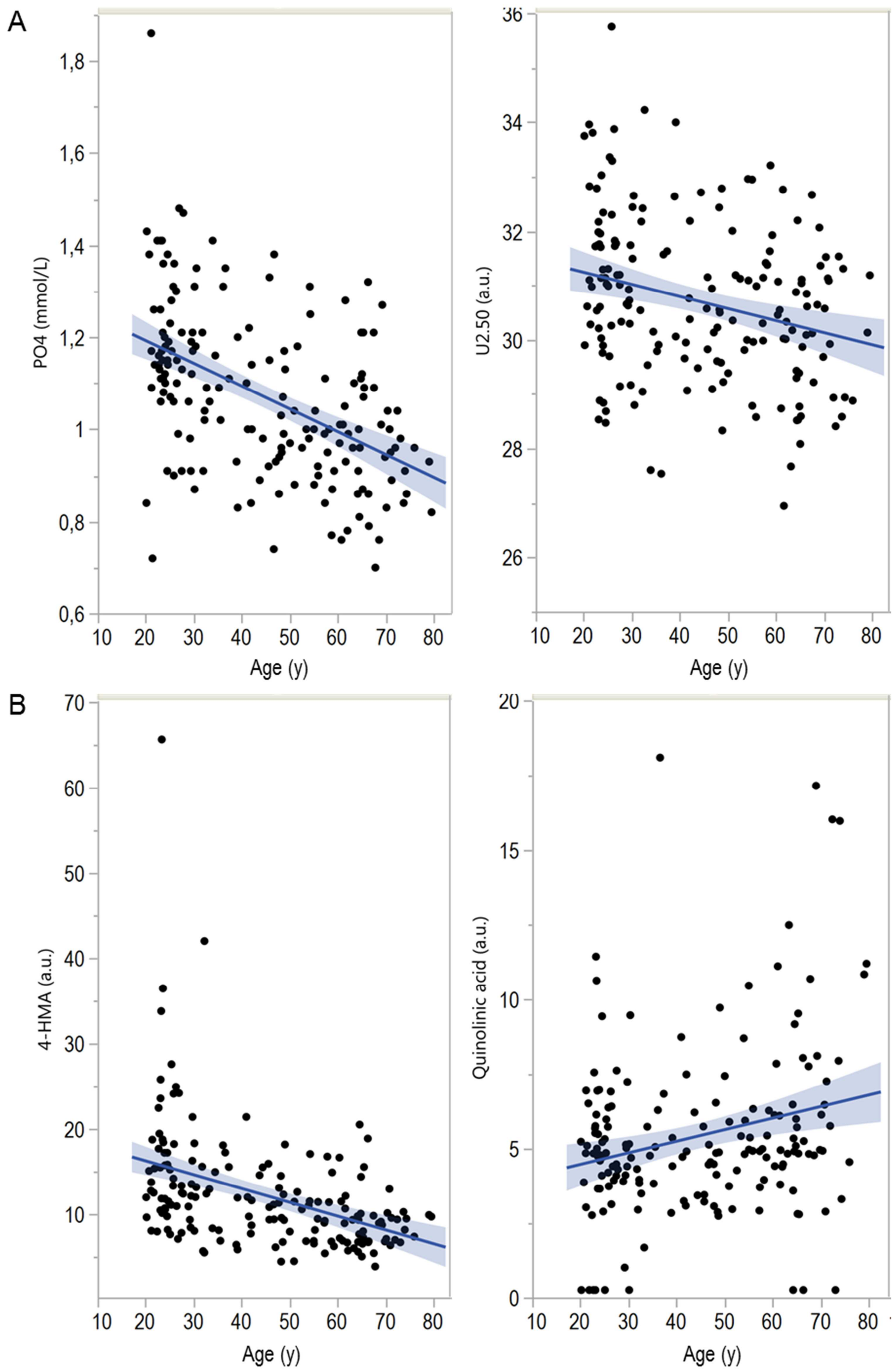

S2 Fig.: Concentrations of selected metabolites contained in metabolite patterns important for prediction of age in male study participants versus age. Plots are shown for (A) plasma and (B) urine metabolites of male study participants with mean rank 1 (left panel) and 25 (right panel). PO4, phosphate; U2.50, unknown NMR analyte at 2.5 ppm; 4-HMA, 4-hydroxymandelic acid; a.u., arbitrary units.
