# Supplementary material for: Metabolite patterns predicting sex and age in participants of the Karlsruhe Metabolomics and Nutrition (KarMeN) study
Source: PLoS One. 2017 Aug 16;12(8):e0183228. doi: 10.1371/journal.pone.0183228 (PMC5558977; doi:10.1371/journal.pone.0183228)
Supplement: S3 Fig — (PDF) [file pone.0183228.s003.pdf]

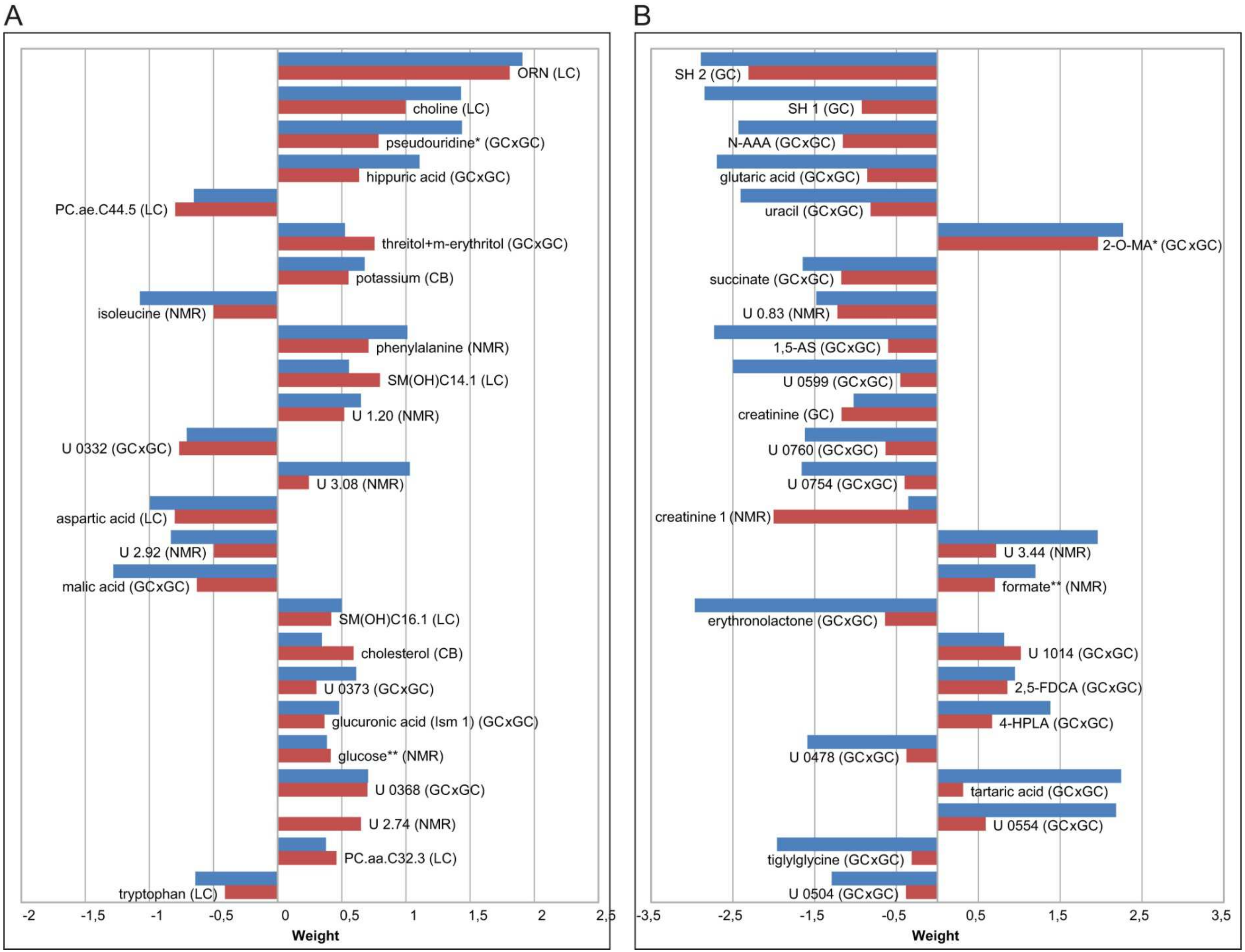
A
ORN (LC)
choline (LC)
pseudouridine* (GCxGC)
hippuric acid (GCxGC)
PC.ae.C44.5 (LC)
threitol+m-erythritol (GCxGC)
potassium (CB)
isoleucine (NMR)
phenylalanine (NMR)
SM(OH)C14.1 (LC)
U 1.20 (NMR)
U 0332 (GCxGC)
U 3.08 (NMR)
aspartic acid (LC)
U 2.92 (NMR)
malic acid (GCxGC)
SM(OH)C16.1 (LC)
cholesterol (CB)
U 0373 (GCxGC)
glucuronic acid (lsm 1) (GCxGC)
glucose** (NMR)
U 0368 (GCxGC)
U 2.74 (NMR)
PC.aa.C32.3 (LC)
tryptophan (LC)
-2
-1,5
-1
-0,5
0
0,5
1
1,5
2
2,5
Weight
B
SH 2 (GC)
SH 1 (GC)
N-AAA (GCxGC)
glutaric acid (GCxGC)
uracil (GCxGC)
2-O-MA* (GCxGC)
succinate (GCxGC)
U 0.83 (NMR)
1,5-AS (GCxGC)
U 0599 (GCxGC)
creatinine (GC)
U 0760 (GCxGC)
U 0754 (GCxGC)
creatinine 1 (NMR)
U 3.44 (NMR)
formate** (NMR)
erythronolactone (GCxGC)
U 1014 (GCxGC)
2,5-FDCA (GCxGC)
4-HPLA (GCxGC)
U 0478 (GCxGC)
tartaric acid (GCxGC)
U 0554 (GCxGC)
tiglylglycine (GCxGC)
U 0504 (GCxGC)
-3,5
-2,5
-1,5
-0,5
0,5
1,5
2,5
3,5
Weight

S3 Fig.: Metabolite patterns for the prediction of age in women. Top 25 metabolites important for the correct prediction of age of the female KarMeN study participants in all algorithms applied on plasma (A) and 24 h urine (B) metabolite profiles. Positive and negative weights favor older and younger age, respectively. Patterns are shown for linear SVM (blue bars) and glmnet (red bars) only, since PLS only yields positive values. Metabolites are sorted according to "mean rank" of all three algorithms. Analytical methods from which metabolites stem are denoted in parentheses, with CB clinical biochemistry; GC GC-MS; GC×GC GC×GC-MS; LC LC-MS; NMR nuclear magnetic resonance.

* Tentatively identified using the NIST2011 library solely based on mass spectral similarity.

** Signal possibly includes other metabolites.

Abbreviations: CB clinical biochemistry; LC LC-MS; GC GC-MS; GC×GC GC×GC-MS; ORN ornithine; U unknown; SH2 sedoheptulose (isomer 2); SH1 sedoheptulose (isomer 1); N-AAA N-acetylaspartic acid; 2-O-MA 2-*O*-methylascorbate; 1,5-AS 1,5-anhydro-D-sorbitol; 2,5-FDCA 2,5-furandicarboxylic acid; 4-HPLA 4-hydroxyphenyllactic acid
