# Supplementary material for: Metabolite patterns predicting sex and age in participants of the Karlsruhe Metabolomics and Nutrition (KarMeN) study
Source: PLoS One. 2017 Aug 16;12(8):e0183228. doi: 10.1371/journal.pone.0183228 (PMC5558977; doi:10.1371/journal.pone.0183228)
Supplement: S4 Fig — (PDF) [file pone.0183228.s004.pdf]

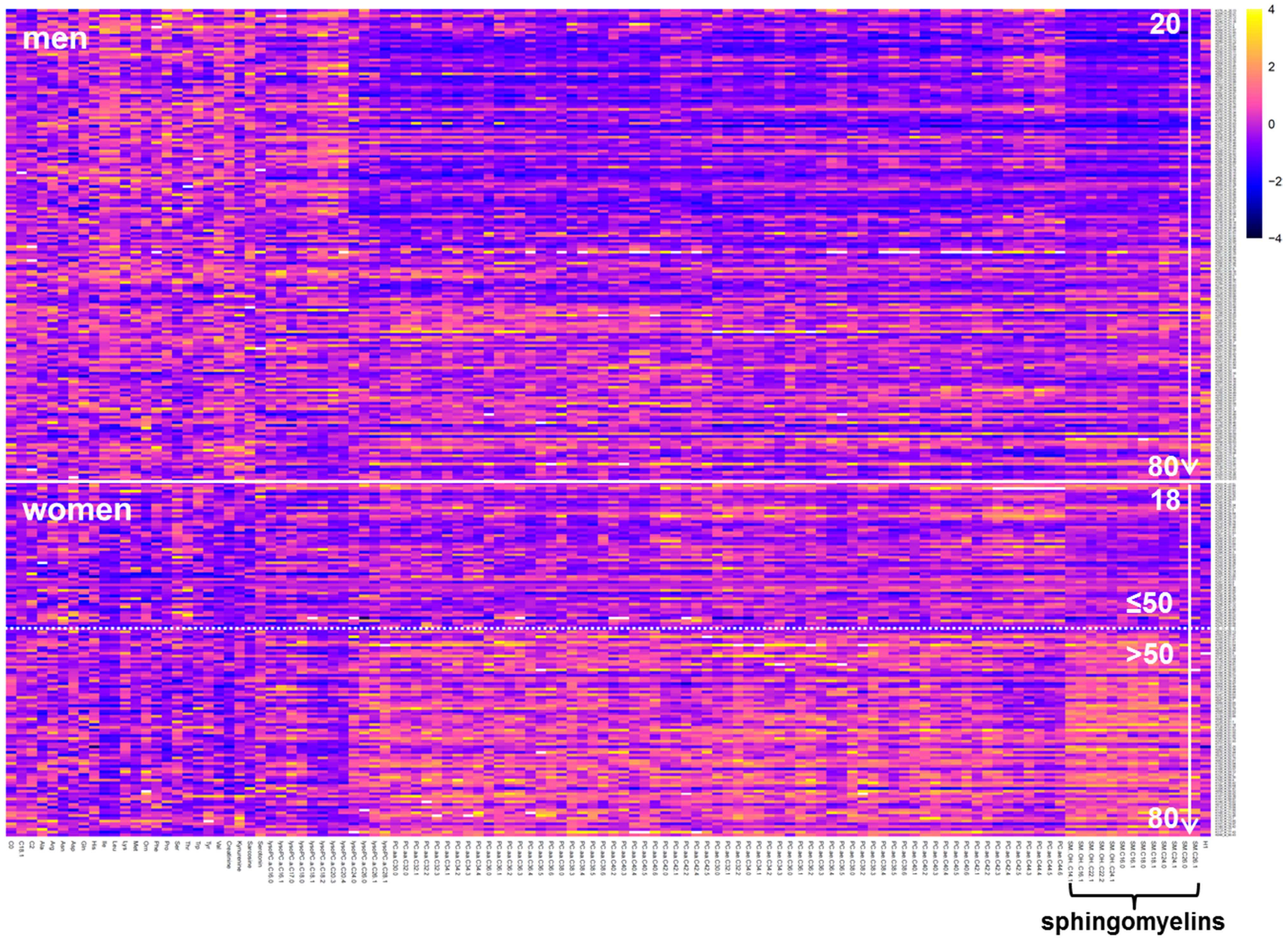

S4 Fig.: Heatmap of metabolites from Biocrates platform, sorted according to sex and age. Metabolites are plotted in columns, study participants in rows; men from young to old age in the upper part, women from young to old age in the bottom part. Most of the lipids are generally higher in concentration in women than in men. Concentrations increase relatively continuously in men, whereas in women a sudden increase can be observed around the age of 50 (see dotted line).
