# Supplementary material for: Metabolite patterns predicting sex and age in participants of the Karlsruhe Metabolomics and Nutrition (KarMeN) study
Source: PLoS One. 2017 Aug 16;12(8):e0183228. doi: 10.1371/journal.pone.0183228 (PMC5558977; doi:10.1371/journal.pone.0183228)
Supplement: S5 Fig — (PDF) [file pone.0183228.s005.pdf]

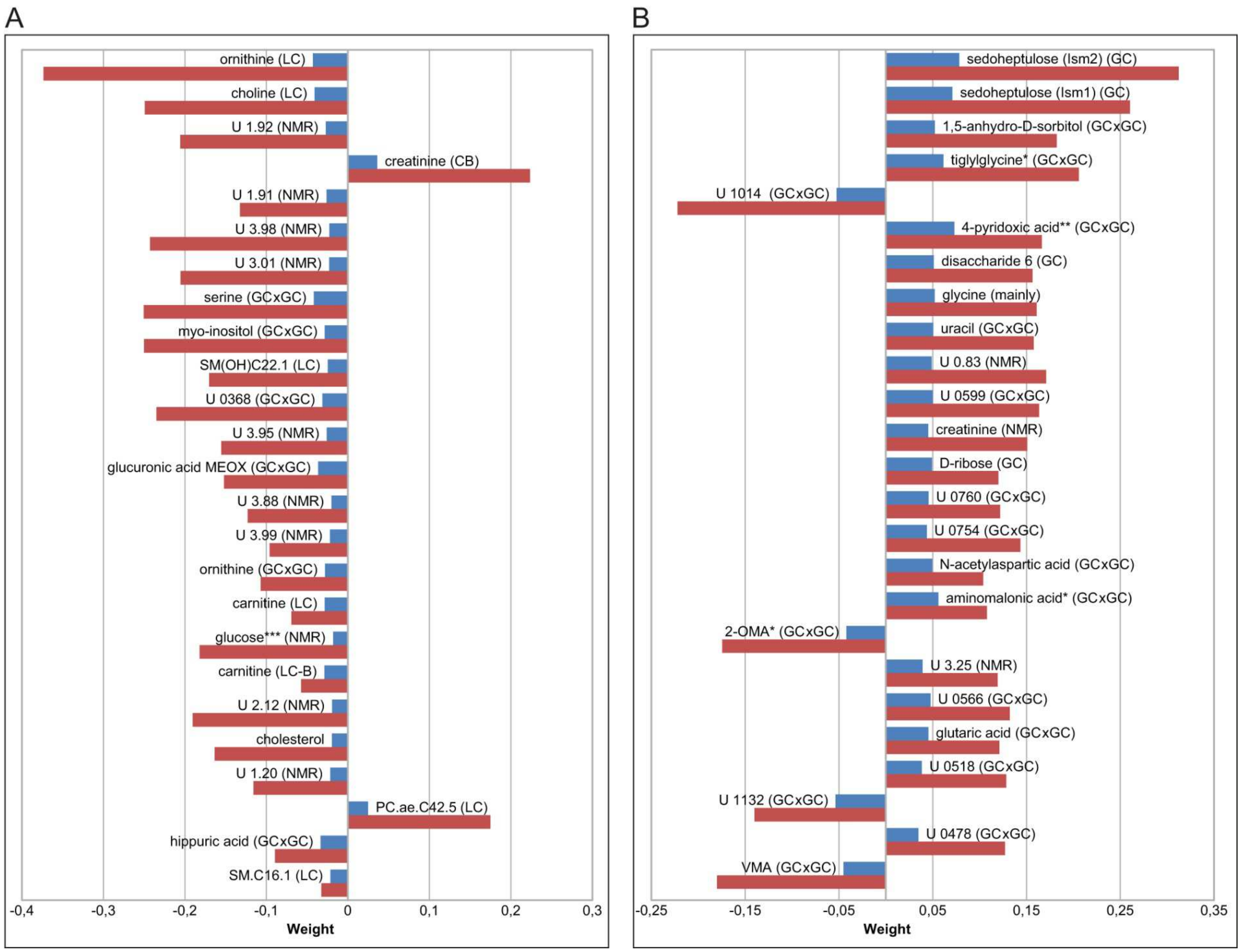
A
ornithine (LC)
choline (LC)
U 1.92 (NMR)
creatinine (CB)
U 1.91 (NMR)
U 3.98 (NMR)
U 3.01 (NMR)
serine (GCxGC)
myo-inositol (GCxGC)
SM(OH)C22.1 (LC)
U 0368 (GCxGC)
U 3.95 (NMR)
glucuronic acid MEOX (GCxGC)
U 3.88 (NMR)
U 3.99 (NMR)
ornithine (GCxGC)
carnitine (LC)
glucose*** (NMR)
carnitine (LC-B)
U 2.12 (NMR)
cholesterol
U 1.20 (NMR)
PC.ae.C42.5 (LC)
hippuric acid (GCxGC)
SM.C16.1 (LC)
-0,4
-0,3
-0,2
-0,1
0
0,1
0,2
0,3
Weight
B
sedoheptulose (Ism2) (GC)
sedoheptulose (Ism1) (GC)
1,5-anhydro-D-sorbitol (GCxGC)
tiglylglycine* (GCxGC)
U 1014 (GCxGC)
4-pyridoxic acid** (GCxGC)
disaccharide 6 (GC)
glycine (mainly)
uracil (GCxGC)
U 0.83 (NMR)
U 0599 (GCxGC)
creatinine (NMR)
D-ribose (GC)
U 0760 (GCxGC)
U 0754 (GCxGC)
N-acetylaspartic acid (GCxGC)
aminomalonic acid* (GCxGC)
2-OMA* (GCxGC)
U 3.25 (NMR)
U 0566 (GCxGC)
glutaric acid (GCxGC)
U 0518 (GCxGC)
U 1132 (GCxGC)
U 0478 (GCxGC)
VMA (GCxGC)
-0,25
-0,15
-0,05
0,05
0,15
0,25
0,35
Weight

S5 Fig.: Metabolite patterns for the prediction of menopausal status in women. Top 25 metabolites important for the correct prediction of menopause of the female KarMeN study participants in all algorithms applied on plasma (A) and 24 h urine (B) metabolite profiles. Positive and negative weights favor pre- and postmenopausal status, respectively. Patterns are shown for linear SVM (blue bars) and glmnet (red bars) only, since PLS only yields positive values.Metabolites are sorted according to "mean rank" of all three algorithms. Analytical methods from which metabolites stem are denoted in parentheses, with CB clinical biochemistry; GC GC-MS; GC×GC GC×GC-MS; LC LC-MS; NMR nuclear magnetic resonance.

* Tentatively identified using the NIST2011 library solely based on mass spectral similarity.

** Identified using the FIEHN library based on mass spectral similarity and retention index.

*** Signal possibly includes other metabolites.

Abbreviations: U unknown; ism isomer; 2-O-MA 2-*O*-methylascorbate; VMA vanillylmandelic acid
