# Supplementary material for: Metabolite patterns predicting sex and age in participants of the Karlsruhe Metabolomics and Nutrition (KarMeN) study
Source: PLoS One. 2017 Aug 16;12(8):e0183228. doi: 10.1371/journal.pone.0183228 (PMC5558977; doi:10.1371/journal.pone.0183228)
Supplement: S2 Table — (PDF) [file pone.0183228.s007.pdf]

S2 Table: Prediction of menopausal status in female study participants.

**Matrix**	**Algorithm**	**Accuracy % (total) n=129**	**Accuracy % (post) n=73**	**Accuracy % (pre) n=56**
Plasma	SVMlinear	87	88.3	85.2
	glmnet	88	89.7	85.7
	PLS	89.1	89.4	88.5
Urine	SVMlinear	82.7	86.9	77.3
	glmnet	83.7	88.8	77.2
	PLS	85.4	88.5	81.4
Plasma and Urine	SVMlinear	90.9	94.9	85.3
	glmnet	89.9	92.9	86
	PLS	89.8	93.4	84.7
