# Supplementary material for: Metabolite patterns predicting sex and age in participants of the Karlsruhe Metabolomics and Nutrition (KarMeN) study
Source: PLoS One. 2017 Aug 16;12(8):e0183228. doi: 10.1371/journal.pone.0183228 (PMC5558977; doi:10.1371/journal.pone.0183228)
Supplement: S1 File — (DOCX) [file pone.0183228.s009.docx]

**Supplemental information**

# Additional methods

# Chemicals for GC- and GC×GC-MS-based analyses

Methanol (GC grade) was purchased from Merck (Darmstadt, Germany). Heptane (>99.0 %) and pyridine (>99.5 %) were from Carl Roth (Karlsruhe, Germany). *N*-Methyl-*N*-(trimethylsilyl)trifluoroacetamide (MSTFA) with 1 % trimethylchlorosilane (TMCS) was supplied by Macherey-Nagel (Düren, Germany). *O*-Methoxylamine hydrochloride was purchased from Chemos (Regenstauf, Germany). Internal standards used for untargeted GC×GC-MS analysis were from Sigma-Aldrich (Steinheim, Germany), Carl Roth (Karlsruhe, Germany), ABCR (Karlsruhe, Germany), Merck (Darmstadt, Germany), TCI (Zwijndrecht, Belgium), Alfa-Aesar (Ward Hill, USA), and ChromaDex (Irvine, USA). All standards had a purity of at least 95 %.

Supelco 37 Component FAME Mix (TraceCERT® grade) was purchased from Altmann Analytik GmbH & Co. KG (Gablingen, Germany). Methyl 9(Z),11(E)-octadecadienoate and methyl 10(E),12(Z)-octadecadienoate (purities >98 %) were obtained from BIOTREND Chemikalien GmbH (Cologne, Germany). All other fatty acid methyl ester reference standards and tritridecanoin were obtained from Larodan Fine Chemicals AB (Solna, Sweden) in a purity of at least 97 %. All solvents used were of GC grade and all other chemicals were of analytical grade.

# Untargeted GC×GC-MS analysis of plasma and urine samples

## Composition of the internal standard solutions used for the untargeted GC×GC-MS analyses

| Internal standard | Concentration (µmol/L) | |
| --- | --- | --- |
|  | Urine | Plasma |
| 1-*O*-Methyl-2-deoxy-d-ribose | 100 | 200 |
| 2-Deoxy-d-glucose | --- | 100 |
| 3-*O*-Methylglucose | 50 | 100 |
| Phenyl-β-d-glucopyranoside | 100 | --- |
| d-Pinitol | 50 | 50 |
| *N*-Methyl-l-serine | --- | 200 |
| *p*-Chloro-l-phenylalanine | 100 | 100 |
| Hexylamine | 100 | --- |
| 2-Amino-6-chloropurine | 150 | --- |
| 2-(4-Chlorophenyl)-ethylamine | 50 | 200 |
| 2-Chlorophenylacetic acid | 100 | 200 |
| 5-Chlorosalicylic acid | 100 | 200 |
| 5-Bromo-2,4-dihydroxybenzoic acid | 50 | 100 |
| *trans*-3,4-(Methylenedioxy)cinnamic acid | 100 | --- |
| 5-Bromo-2-hydroxyhippuric | 100 | 200 |

## Preparation of urine samples

Sample preparation was done as described recently by Weinert et al. [[1](#_ENREF_1)], with the following modifications: The composition of internal standard solutions was slightly adapted according to the requirements of the respective matrix (see section 2.1) while the added volume was always 20 µL. Urine samples were diluted to adjust osmolality to 60 mosm/kg before evaporation. After evaporation, 20 µL of methanol were added and the samples dried again for 30-45 min to remove remaining traces of water.

## Preparation of plasma samples

Firstly, 20 µL of an internal standard solution (see section 2.1) and 40 µL of plasma were placed in a 1.5 mL Eppendorf reaction tube. Secondly, proteins were removed by rapid addition of 150 µL of ice-cold methanol and centrifugation for 10 min at 16,100 x g and 4 °C. Afterwards, 120 µL of the supernatant were transferred into a fresh Eppendorf tube, 700 µL of *tert*-butyl methyl ether were added and samples were vigorously mixed. Phase separation was induced by addition of 50 µL of water. After renewed mixing and a short spin-down, the upper lipid-containing TBME layer was discarded and the lower aqueous phase was transferred into a GC screw-top vial with 200 µL inserts. The Eppendorf tube was rinsed with 30 µL of methanol and the wash solution was combined with the aqueous phase in the vial. Evaporation was done in a speed-vac (1 h, 40 °C, p < 1 mbar). To remove remaining traces of water, 10 µL of methanol were added and samples dried again for 15 min. Methoximation was carried out by addition of 20 µL of methoxylamine-hydrochloride in pyridine (20 mg/mL) at 40 °C for 1 h under shaking. Afterwards, trimethylsilylation was conducted using 40 µL of MSTFA with 1 % TMCS (75 °C, 1.5 h). Solvent blanks were created and processed as in case of the urine samples, including the final addition of the retention index marker mixture (see [[1](#_ENREF_1)]).

## GC×GC-MS instrument and software

| **Instrument component / software** | **Name** | **Manufacturer** |
| --- | --- | --- |
| Gas chromatograph | GC-2010 | Shimadzu Corp, Kyoto, Japan |
| Mass spectrometer | QP2010 Ultra | Shimadzu Corp, Kyoto, Japan |
| Auto sampler | AOC-5000 | Shimadzu Corp, Kyoto, Japan |
| PTV Injector | OPTIC-4 | GL Sciences, Eindhoven, The Netherlands |
| Modulator | ZX2 | ZOEX Corp., Houston, USA |
| GCMS instrument software | GCMS Solution 4.11 | Shimadzu Corp, Kyoto, Japan |
| PTV software | Evolution Workstation 4.1 | GL Sciences, Eindhoven, The Netherlands |
| GC×GC visualization software | ChromSquare 2.1 | Chromaleont Srl, Messina, Italy |

## Method parameters and consumables

| **Parameter** | **Setting / value** |
| --- | --- |
| **GC parameters** | |
| Carrier gas | Helium |
| GC mode | Constant velocity |
| Initial column head pressure | Urine: 90 kPa; Plasma: 60 kPa |
| Liner type | Deactivated split liner with quartz wool |
| ^1^D column | Restek Rxi-5SilMS; ^1^L = 15 m plus 5 m of an integrated pre-column; ^1^d_c_ = 0.25 mm; ^1^d_f_ = 0.25 µm |
| ^2^D column | SGE BPX50; ^2^L_total_ = 2.6 m, including a “separation segment” of ^2^L_sep_= 1.1 m; ^2^d_c_ = 0.15 mm, ^2^d_f_ = 0.15 µm |
| Column connector | SilTite MiniUnion (SGE) |
| GC temperature ramp (urine) | 90 °C → 2 °C/min → 100 °C → 3.75 °C/min → 160 °C → 4 °C/min → 200 °C → 3.5 °C/min → 268 °C → 25 °C/min → 320 °C (1.49 min). Run time: 54 min. |
| GC temperature ramp (plasma) | 90 °C → 2 °C/min → 100 °C → 3.5 °C/min → 240 °C → 5 °C/min → 280 °C → 40 °C/min → 320 °C (2.50 min). Run time: 56.5 min. |
| Injection mode | Cold split |
| Injection volume | Urine: 1.2 µL (evaluation samples: 1.6 µL)  Plasma: 1.0 µL (evaluation samples: 1.5 µL) |
| Split ratio | Urine: 1:8; Plasma: 1:10 |
| PTV temperature ramp | 90 °C → 60 °C/s→ 280 °C, hold until end of run |
| Interface temperature | 320 °C |
| **Modulation parameters** | |
| Modulator type | Cryogenic, air-based, loop-type |
| Modulation period (P_M_) | 6 s (urine), 4.5 s (plasma) |
| Cold jet temperature | -90 °C |
| Hot jet temperature | Programmed stepwise, at least 50 °C above oven temperature |
| Hot jet duration | 375 ms |
| **MS parameters** | |
| Ion source temperature | 200 °C |
| Ionization mode | EI (70 eV) |
| MS Mode | Scan |
| Scan speed | 20.000 u/s |
| Scan range | m/z 60-550 |
| Event time | 30 ms |
| Data acquisition frequency | 33 s^-1^ |

## GC×GC-MS analysis of urine and plasma samples

In order to enable tight quality control and to correct drift and offset effects [1], pooled quality control (QC) samples, prepared from a representative subset of the study samples, were used and always injected twice. The effect of the QC sample-based drift correction was controlled using additional QC samples (termed “evaluation samples”) which were prepared like the QC samples but of which a higher volume was injected (see section 2.5.). Each day, 14 study samples, four QC samples, the evaluation sample and a solvent blank were prepared and analysed according to a standardized measurement protocol. After the daily blank, the analysis of the matrix samples was initiated by the first QC twin injection, followed by five study samples, the next QC twin injection and so on. Every day, the evaluation sample replaced one of the study samples at a randomized position. At the beginning of each week, the liner was changed and the qMS was tuned, followed by six to eight equilibration runs. The injector septum was replaced after approx. 90 runs.

## Evaluation of GC×GC-MS data sets

After automatic processing, the quality of the GC×GC-qMS data sets was evaluated in two steps:

At first, the integrity of the QC and study sample runs was assessed based on the reliable internal standards (mean intra-day RSD: 3.8-10.4 % (urine), 3.6-10.0 % (plasma)). QC and study samples for which the signal intensities of these internal standards deviated (on average) by more than +/- 20 % from the daily median of all samples were excluded. Secondly, the analytes detectable in at least 75 % of the study samples (plasma) were closely inspected in order to remove i) known artefacts, ii) remaining sections of noise bands not eliminated during denoising, iii) analytes affected by coelution and iv) analytes exceeding mean intra-day repeatability limits (RSD ≤ 20 %; in case of trace analytes and intra-day trends, an RSD of up to 30 % was accepted as described by Dunn et al. [[2](#_ENREF_2)]).

# Semi-targeted GC-MS analysis of sugar species in urine samples

## Composition of the internal standard solution.

| Internal standard | Concentration (µmol/L) |
| --- | --- |
| 1-*O*-Methyl-2-deoxy-d-ribose | 100 |
| Phenyl-β-d-glucopyranoside | 100 |
| d-Pinitol | 50 |

## Preparation of urine samples

Sample preparation was done as described in section 2.2. The composition of internal standard solutions is given in section 3.1, the added volume was 20 µL. Methoximation was carried out for 30 min at 70 °C and 1000 rpm and trimethylsilylation for 1 h at 75 °C without shaking, using the same reagent volumes.

## GC-MS instrument and software

| **Instrument component** | **Name** | **Manufacturer** |
| --- | --- | --- |
| Gas chromatograph | GC-2010 | Shimadzu Corp, Kyoto, Japan |
| Mass spectrometer | QP2010 Ultra | Shimadzu Corp, Kyoto, Japan |
| Auto sampler | AOC-5000 | Shimadzu Corp, Kyoto, Japan |
| PTV Injector | OPTIC-4 | GL Sciences, Eindhoven, The Netherlands |
| GCMS instrument software | GCMS Solution 4.11 | Shimadzu Corp, Kyoto, Japan |
| PTV software | Evolution Workstation 4.1 | GL Sciences, Eindhoven, The Netherlands |

## Method parameters and consumables

| **Parameter** | **Setting / value** |
| --- | --- |
| **GC parameters** | |
| Carrier gas | Helium |
| GC mode | Constant pressure (256 kPa) |
| Liner type | Deactivated split liner with quartz wool |
| Column | Restek Rxi-5SilMS; L = 60 m plus 10 m of an integrated pre-column; d_c_ = 0.25 mm; d_f_ = 0.25 µm |
| GC temperature ramp | 150 °C → 4 °C/min → 180 °C → 7 °C/min → 200 °C → 2.25 °C/min → 235 °C → 10 °C/min → 270 °C → 5.25 °C/min → 310 °C → 20 °C/min → 330 °C (3,97 min). Run time: 42 min. |
| Injection mode | Cold split |
| Injection volume | 1.2 µL (evaluation samples: 1.6 µL) |
| Split ratio | 1:5 |
| PTV temperature ramp | 90 °C → 60 °C/s→ 280 °C, hold until end of run |
| Interface temperature | 320 °C |
| **MS parameters** | |
| Ion source temperature | 200 °C |
| Ionization mode | EI (70 eV) |
| MS Mode | Scan/SIM |
| Scan speed | 20.000 u/s |
| Scan range | m/z 60-600 |
| Event time (scan) | 40 ms |
| SIM time frame 1 (7.00-15.95 min) | Event time: 500 ms; m/z 147.05, 204.10, 217.10, 307.15, 319.20, 117.00, 160.10, 292.15, 277.20, 333.10, 245.00, 218.00, 329.00, and 314.00 |
| SIM time frame 2 (15.95 - 21.50 min) | Event time: 600 ms; m/z 147.05, 204.10, 217.10, 307.15, 319.20, 117.00, 160.10, 318.20, 205.10, 333.10, 361.15, 220.00, 202.10, 260.00, 345.00, 255.00, 156.00, 206.00, and 105.00 |
| SIM time frame 3 (21.50-32.20 min) | Event time: 580 ms; m/z 147.05, 204.10, 217.10, 307.15, 319.20, 318.20, 205.10, 333.10, 299.00, 202.10, 361.15, 441.00, 382.00, and 357.00 |
| SIM time frame 4 (32.20-41.99 min) | Event time: 330 ms; m/z 147.05, 204.10, 217.10, 307.15, 319.20, and 361.15 |

## GC-MS analysis of urine samples

The semi-targeted analysis of urine samples was done as described in section 2.6 with minor adjustments. Due to the shorter run-time each day, 23 study samples and the evaluation sample were measured in four groups of six samples framed by five doubly injected QC samples.

## Integration and identification parameters

| **parameter** | **setting/value** |
| --- | --- |
| slope | 100/min |
| width | 1 s |
| drift | 0/min |
| T.DBL | 1000 min |
| minimal area | 0 |
| smoothing | none |
| default bandtime | 0.1 min |
| spectrum confirmation | use ratios of target and reference ions* |
| reference ion mode | absolute |
| ratio deviation allowance | 30 %* |

* In some cases it became necessary to use a higher allowance, to use only one or no reference ion because of overlapping peaks with similar MS spectra.

## Evaluation of the semi-targeted GC-MS data set

Automatic integration data was checked manually with respect to peak assignment and occurrence of coelution. Reasons for wrongly assigned or not at all assigned peaks were inspected and reevaluated according to the following points: i) for overlapping peaks with similar MS spectra a higher deviation of reference ion rations was accepted ii) because of similar retention times and MS spectra wrongly assigned peaks were correctly assigned by hand. Integrity of the QC and study sample runs was assessed as described for the GC×GC-qMS data sets (mean intra-day RSD: 2.6-8.2 %).

# Targeted GC-MS analysis of fatty acids in plasma samples

## Preparation of plasma samples

20 μL of plasma were added to PTFE screw capped Pyrex tubes containing 7.5 μg of each, C19:0 methyl ester (internal standard) and tritridecanoin (internal derivatization control). Derivatization was carried out with 2 mL of methanolic acetylchloride (10 %) and 500 μL of n-hexane at room temperature and 700 rpm in a rotator mixer overnight. Following addition of 5 mL of 6 % aqueous potassium carbonate solution and centrifugation at 2000 rpm, 350 μL of the n-hexane layer were dried over sodium sulfate in a crimped 500 μL-GC vial. After centrifugation at 2000 rpm, 200 μL of the dried n-hexane extract were transferred into a 500 μL-GC crimp vial for GC-MS analysis. Two daily blank samples were prepared accordingly, using 20 μL of water instead of plasma.

## GC-MS instrument and software

| **Instrument component** | **Name** | **Manufacturer** |
| --- | --- | --- |
| Gas chromatograph | GC-2010 | Shimadzu Corp, Kyoto, Japan |
| Mass spectrometer | QP2010 Ultra | Shimadzu Corp, Kyoto, Japan |
| Auto sampler | AOC-20s | Shimadzu Corp, Kyoto, Japan |
| Auto injector | AOS-20i | Shimadzu Corp, Kyoto, Japan |
| GCMS instrument software | GCMS Solution 4.11 | Shimadzu Corp, Kyoto, Japan |

## Method parameters and consumables

| **Parameter** | **Setting / value** |
| --- | --- |
| **GC parameters** | |
| Carrier gas | Helium |
| GC mode | Constant velocity (40 cm/s) |
| Liner type | Intermediate polarity deactivated 3.5 mm ID straight liner with deactivated glass wool |
| Column | SGE BPX90; L = 60 m; d_c_ = 0.25 mm; d_f_ = 0.25 µm |
| GC temperature ramp | 120°C (5 min) → 2°C/min → 150°C (12 min) → 2°C/min → 180°C (2 min) → 2°C/min → 190°C (3 min) → 5°C/min → 250°C (10 min). Run time: 79 min. |
| Injection mode | Hot split (260 °C) |
| Injection volume | 1.0 µL |
| Split ratio | 1:50 |
| Interface temperature | 260 °C |
| **MS parameters** | |
| Ion source temperature | 200 °C |
| Ionization mode | EI (70 eV) |
| MS Mode | SIM |
| Event time | 30 ms |
| SIM window* 1 (3.2 – 16.2 min) | m/z 74.0, 87.0, 143.0, 55.0, 69.0 |
| SIM window 2 (16.2 – 22.2 min) | m/z 55.0, 69.0, 74.0, 236.0, 87.0, 143.0 |
| SIM window 3 (22.2 – 24.0 min) | m/z 55.0, 69.0, 83.0, 264.0 |
| SIM window 4 (24.0 – 28.0 min) | m/z 67.0, 81.0, 95.0, 294.0, 74.0, 87.0, 143.0, 55.0 |
| SIM window 5 (28.0 – 30.5 min) | m/z 79.0, 67.0, 80.0, 74.0, 87.0, 143.0 |
| SIM window 6 (30.5 – 34.0 min) | m/z 79.0, 67.0, 95.0, 108.0, 55.0, 69.0, 83.0, 292.0, 81.0, 294.0 |
| SIM window 7 (34.0 – 37.5 min) | m/z 79.0, 91.0, 93.0, 74.0, 87.0, 143.0, 67.0, 81.0, 322.0 |
| SIM window 8 (37.5 – 40.5 min) | m/z 79.0, 67.0, 80.0, 320.0, 74.0, 87.0, 143.0 |
| SIM window 9 (40.5 – 42.5 min) | m/z 55.0, 79.0, 95.0, 320.0, 106.0, 80.0, 91.0, 69.0 |
| SIM window 10 (42.5 – 46.0 min) | m/z 79.0, 93.0, 67.0, 74.0, 87.0, 143.0, 81.0, 82.0, 350.0, 91.0 |
| SIM window 11 (46.0 – 48.2 min) | m/z 74.0, 87.0, 382.0 |
| SIM window 12 (48.2 – 51.0 min) | m/z 79.0, 80.0, 67.0, 55.0, 69.0, 83.0, 348.0, 91.0 |
| SIM window 13 (51.0 – 60.0 min) | m/z 79.0, 91.0, 67.0 |

*SIM windows were updated as needed after maintenance procedures.

## GC-MS analysis

Individual FAMEs were detected in SIM mode using the most intense fragments for quantification and two additional ions as qualifiers. In only a few cases, application of other, selective quantifier ions was required due to insufficient chromatographic separation. Weekly external calibrations with C19:0 methyl ester as internal standard were used for quantification. Each day, plasma QC samples were run prior to the first sample, after every seventh sample and after the last sample, respectively. Coefficients of variation calculated from 70 QC samples analyzed over 7 weeks were <4 %, <10 %, and <16 %, for FAMEs at high (>5 ng/μL injected sample), medium (0.2‑5 ng/μL), and low (<0.2 ng/μL) concentration levels, respectively, with the exception of C10:0, C12:0 and C20:0, which were present at low levels and showed coefficients of variation from 19-34 %.

# Targeted LC-MS metabolite profiling using the Absolute IDQ™ p180 kit

## Preparation of plasma samples

A 20 µL plasma aliquot was used for each extraction. A protein precipitation and subsequent derivatization with phenyl isothiocyanate (PITC) was performed according to the manufacturer’s protocol (UM-P180-ABSCIEX-7, Biocrates).

## LC-MS instrument and software

PITC derivatives of amino acids and biogenic amines were separated on an Agilent Zorbax Eclipse XDB-C18 column (3x100 mm, 3.5 µm) equipped with a Phenomenex Security Guard (C18, 4.0 x3.0 mm) prior to MS-detection. The analytical system comprised an API 5500 Q-Trap mass spectrometer (AB Sciex) coupled to a Shimadzu Nexera UHPLC-system. Phosphatidylcholines and sphingomyelins were analyzed by flow injection analysis (FIA). The Absolute IDQ kit provides optimized MS instrument settings and compound parameters.{Romisch-Margl, 2012 #933} System control and data acquisition was carried out with Analyst 1.5.2. software. Quantification and data evaluation was done with the MetIDQ software (version 4.5.2).

## Reliability of results

The Absolute IDQ™ p180 kit is validated according to FDA guidelines. However, to ensure reliability of our results, QC-samples included in the kit were injected ten times distributed between the study samples. Additionally, to obtain QC samples closely related to study samples (similar matrix and concentration), we also used pooled study plasma like for all other analytical methods. Six replicates of this study-specific QC sample were extracted per plate and evenly distributed amongst the study samples and each injected as duplicates. Analytes were included in further data analysis if study-specific QC samples of all plates met the following criteria for repeatability: RSD ≤ 20 % (quantitative analytes), RSD ≤ 25 % (semi-quantitative analytes).

# Targeted LC-MS analysis of methylated amino compounds

## Chemicals

Acetonitrile (LC-MS grade) was from VWR (Darmstadt, Germany). Ammonium formate (HPLC grade) and formic acid (LC-MS grade) were from Sigma-Aldrich (Steinheim, Germany). Standards for amino compounds and their deuterated isotopologues were from TRC (Toronto, Canada) and supplied by Biozol (Eching, Germany).

## Preparation of plasma samples

Sample preparation (10 µL plasma) was performed by protein precipitation and dilution with acetonitrile 1:10, and subsequent centrifugation before direct transfer to LC vials without evaporation step [[3](#_ENREF_3)]. Matrix-adapted calibrators and controls were produced by spiking of human plasma. For calibration, plasma aliquots were freshly spiked for each sequence. Controls were spiked at three levels and aliquots were stored at -20 °C for subsequent use. The same raw plasma for spiking was used throughout all sequences. Standard addition was performed to quantify the analyte amounts already present in the raw plasma before spiking. By this way, the true values of the spiked calibrators (natural background + spike) were determined and subsequently used for building the calibration curve for unknown study samples.

## LC-MS instrument and software

| **Instrument component** | **Name** | **Manufacturer** |
| --- | --- | --- |
| Liquid chromatograph | Acquity H-Class | Waters |
| Mass spectrometer | Xevo TQD | Waters |
| Instrument software | MassLynx SCN855 | Waters |

## Method parameters and consumables

| **Parameter** | **Setting / value** |
| --- | --- |
| **LC parameters** | |
| Column | Acquity BEH Amide, 1.7 µm, 2.1 mm x 100 mm |
| Eluent A | 1: 1 aceonitrile + 50 mM aqueous ammonium formate, adjusted to pH 3.2 with formic acid |
| Eluent B | aceonitrile + 0.05 % formic acid |
| Gradient | see gradient table |
| Flow rate | 0.6 mL/min |
| Injection volume | 1 µL |
| Column temperature | 30°C |
| Autosampler temperature | 12°C |
| **MS parameters** | |
| Ionization mode | ESI |
| Polarity | positive |
| Scan parameters | see compound table |
| Desolvation temperature | 500°C |
| Desolvation gas | 1000 L/h |
| Cone gas | 50 L/h |
| Source temperature | 150 °C |
| Capillary voltage | 2.0 kV |
| Solvent delay | 1 min |

Gradient table LC:

| **time [min]** | **Eluent A [%]** | **Eluent B [%]** |
| --- | --- | --- |
| 0 | 12 | 88 |
| 0.3 | 26 | 74 |
| 0.8 | 26 | 74 |
| 2.9 | 65 | 35 |
| 2.91 | 12 | 88 |
| 4.50 | 12 | 88 |

Compound table MS:

|  | **Compound Name** | **Parent (m/z)** | **Daughter (m/z)** | **Dwell (s)** | **Cone (V)** | **Collision (V)** |
| --- | --- | --- | --- | --- | --- | --- |
| 1 | TMAO | 76.03 | 58.08 | 0.017 | 35 | 15 |
| 2 | TMAO | 76.03 | 59.07 | 0.017 | 35 | 15 |
| 3 | d9-TMAO | 85.10 | 68.10 | 0.017 | 35 | 15 |
| 4 | Sarcosine | 89.97 | 44.04 | 0.017 | 18 | 10 |
| 5 | d3-Sarcosine | 92.99 | 47.06 | 0.017 | 18 | 10 |
| 6 | Dimethylglycine | 103.97 | 58.08 | 0.017 | 28 | 12 |
| 7 | Choline | 104.03 | 44.94 | 0.017 | 42 | 16 |
| 8 | Choline | 104.03 | 60.00 | 0.017 | 42 | 16 |
| 9 | d6-Dimethylglycine | 110.02 | 64.13 | 0.017 | 28 | 12 |
| 10 | d9-Choline | 113.10 | 69.08 | 0.017 | 42 | 16 |
| 11 | Betaine, Methylcholine | 117.97 | 58.08 | 0.017 | 38 | 12 |
| 12 | Betaine, Methylcholine | 117.97 | 59.07 | 0.017 | 38 | 16 |
| 13 | d3-Betaine | 120.99 | 62.19 | 0.017 | 38 | 16 |
| 14 | Carnitine | 162.01 | 60.00 | 0.017 | 36 | 16 |
| 15 | Carnitine | 162.01 | 102.82 | 0.017 | 36 | 16 |
| 16 | d9-Carnitine | 171.08 | 102.82 | 0.017 | 36 | 16 |

## Integration and identification parameters

Data processing was performed by TargetLynx using the Apex Track algorithm for peak integration. Peaks were smoothed by mean (1-2 times, smoothing width 2) and calibration was linear without weighting.

## Method evaluation

Recoveries were determined by using deuterated analogues and were estimated to 81-93 % (2 levels). Matrix effects were small and matrix-driven signal changes were only -10 % to +20 % depending on the analyte. Precision (intra-day, inter-day) was between 2 % and 8 % (2-3 level), with the exception of sarcosine (10-15 %). The bias (estimate for accuracy) was between -8 % and +10 %, in relation to the calculated spike values. Depending on the analyte, LOQs between 0.4 and 9.4 µM were achieved (criterion: max. 25 % relative standard deviation [RSD]), and the linear range exceeded two orders of magnitude for most analytes.

# Targeted LC-MS analysis of bile acids - reliability of results

To evaluate repeatability during the measurements, study specific QC samples were used as quality control and measured three times within one extraction batch. Repeatability of individual bile acids was between 4.6 % and 13.3 % RSD.

# Untargeted 1D-^1^H-NMR analysis of plasma and urine samples

## Materials

KH_2_PO_4_ and KOH were obtained from Merck (Darmstadt, Germany), Na_2_HPO_4_ x 7H_2_O and HCl were from Sigma (Steinheim, Germany); trimethylsilyl propionic adic-d_4_ (TSP, 98 atom% D), NaN_3_ and D_2_O (99.9 atom% D) were supplied by Aldrich (Steinheim, Germany). All chemicals were analytical grade and used without further purification. NMR tubes were from Duran (supplied by Carl Roth GmbH & Co. KG, Karlsruhe, Germany).

## Preparation and analysis of plasma samples

Plasma samples were mixed in a 1:1 ratio with buffer (75 mM sodium phosphate, 0.08 % (w/v) TSP-d_4_, 6 mM NaN_3_, 20 % D_2_O, pH 7.4), transferred into 5 mm NMR tubes and measured at 310 K on an AVANCE II 600 MHz NMR spectrometer equipped with a ^1^H-BBI probehead with a BACS sample changer (Bruker BioSpin GmbH, Rheinstetten, Germany). 1D spectra were recorded with 36k complex data points, 90.5 receiver gain and 32 scans at 310 K using a CPMG experiment with presaturation for water suppression. An echo time of 3 µs and a prescan delay of 4 s were used. T2 filter was applied with 128 loops. Pulse length was determined automatically by the Bruker AU program pulsecal and presaturation was set corresponding to a 25 Hz pulse. Irradiation frequency for water suppression was optimized once prior to acquisition. Since the sample changer is not cooled and plasma samples are not stable at room temperature, no more than 30 samples were analysed per day. On each measurement day, at least 2 QC samples were analysed at the beginning and the end of the batch.

## Preparation and analysis of urine samples

Urine samples were analysed at 300 K on a Bruker 600 MHz spectrometer (either AVANCE III equipped with a ^1^H,^13^C,^15^N-TCI inversely detected cryoprobe or AVANCE II with ^1^H-BBI room temperature probe (Bruker BioSpin GmbH, Rheinstetten, Germany)) equipped with either SampleXpress or BACS sample changer, respectively, as described in Rist et al. [[4](#_ENREF_4)]. Since urine samples are more stable at room temperature than plasma samples, up to 70 urine samples could be measured per day. At least 3 QC samples were run over the time course of each measurement day.

# Data analysis –nested cross-validation scheme

The inner cross validation (CV) loop was used to find the optimal hyperparameter, while the outer loop was used to assess the accuracy of the prediction. This procedure assures that the performance estimation of the prediction is unbiased because the model is trained and optimized in the inner loop and afterwards the model predicts data in the outer loop on data not seen before. We utilized 10-fold CV repeated 5 times resulting in 5x10-fold CV for inner and outer loop. Performance measures in each run of the outer 5x10-fold CV were averaged, resulting in n=50 iterations. This procedure allows the calculation of unbiased performance estimates of the prediction models. However, it is not possible to get information about how important every variable of the data matrix (analyte) is because in each run of the inner loop the model is slightly different. This is due to the fact that for each run the input data set is different and hence the hyperparameters are chosen to best fit to this data. Theoretically, the hyperparameters could differ in each run. Hence this procedure is only used for performance estimation and afterwards the complete data set is used to train a model whose hyperparameters are set to an average of all the runs of the outer loop of the CV. In case of linear SVM and glmnet negative and positive weights occur, e.g. favoring the male or female class, respectively. In case of PLS positive and negative weight are distributed over multiple components and can only be summarized into a single value by summing up the squared weights from the different components.

**References:**

1. Weinert, C.H., B. Egert, and S.E. Kulling, *On the applicability of comprehensive two-dimensional gas chromatography combined with a fast-scanning quadrupole mass spectrometer for untargeted large-scale metabolomics.* Journal of Chromatography A, 2015. **1405**: p. 156-167.

2. Dunn, W.B., et al., *Procedures for large-scale metabolic profiling of serum and plasma using gas chromatography and liquid chromatography coupled to mass spectrometry.* Nature Protocols, 2011. **6**(7): p. 1060-1083.

3. Weinert, C.H., et al., *The influence of a chronic L-carnitine administration on the plasma metabolome of male Fischer 344 rats.* Molecular Nutrition & Food Research, 2016: p. 1600651-n/a.

4. Rist, M.J., et al., *Influence of Freezing and Storage Procedure on Human Urine Samples in NMR-Based Metabolomics.* Metabolites, 2013. **3**(2): p. 243-58.
